# The Legacy of the TTASAAN Report—Premature Conclusions and Forgotten Promises: A Review of Policy and Practice Part I

**DOI:** 10.3389/fneur.2021.749579

**Published:** 2022-03-28

**Authors:** Dan G. Pavel, Theodore A. Henderson, Simon DeBruin

**Affiliations:** ^1^Pathfinder Brain SPECT Imaging, Deerfield, IL, United States; ^2^The International Society of Applied Neuroimaging (ISAN), Denver, CO, United States; ^3^The Synaptic Space, Inc., Denver, CO, United States; ^4^Neuro-Luminance, Inc., Denver, CO, United States; ^5^Dr. Theodore Henderson, Inc., Denver, CO, United States; ^6^Good Lion Imaging, Columbia, SC, United States

**Keywords:** dementia, traumatic brain injury, seizure, neurotoxicity, depression, bipolar disorder, ADHD, PTSD-posttraumatic stress disorder

## Abstract

Brain perfusion single photon emission computed tomography (SPECT) scans were initially developed in 1970's. A key radiopharmaceutical, hexamethylpropyleneamine oxime (HMPAO), was originally approved in 1988, but was unstable. As a result, the quality of SPECT images varied greatly based on technique until 1993, when a method of stabilizing HMPAO was developed. In addition, most SPECT perfusion studies pre-1996 were performed on single-head gamma cameras. In 1996, the Therapeutics and Technology Assessment Subcommittee of the American Academy of Neurology (TTASAAN) issued a report regarding the use of SPECT in the evaluation of neurological disorders. Although the TTASAAN report was published in January 1996, it was approved for publication in October 1994. Consequently, the reported brain SPECT studies relied upon to derive the conclusions of the TTASAAN report largely pre-date the introduction of stabilized HMPAO. While only 12% of the studies on traumatic brain injury (TBI) in the TTASAAN report utilized stable tracers and multi-head cameras, 69 subsequent studies with more than 23,000 subjects describe the utility of perfusion SPECT scans in the evaluation of TBI. Similarly, dementia SPECT imaging has improved. Modern SPECT utilizing multi-headed gamma cameras and quantitative analysis has a sensitivity of 86% and a specificity of 89% for the diagnosis of mild to moderate Alzheimer's disease—comparable to fluorodeoxyglucose positron emission tomography. Advances also have occurred in seizure neuroimaging. Lastly, developments in SPECT imaging of neurotoxicity and neuropsychiatric disorders have been striking. At the 25-year anniversary of the publication of the TTASAAN report, it is time to re-examine the utility of perfusion SPECT brain imaging. Herein, we review studies cited by the TTASAAN report vs. current brain SPECT imaging research literature for the major indications addressed in the report, as well as for emerging indications. In Part II, we elaborate technical aspects of SPECT neuroimaging and discuss scan interpretation for the clinician.

## Introduction

In 1996, the Therapeutics and Technology Assessment Subcommittee of the American Academy of Neurology (TTASAAN) issued a report regarding the use of single photon emission computed tomography (SPECT) in functional brain imaging (1). Although the TTASAAN report was published in January 1996, it was completed and approved for publication in October of 1994. Consequently, the referenced brain SPECT studies relied upon to derive the conclusions of the TTASAAN report predominately pre-dated the introduction of stabilized radiopharmaceuticals in 1993. In fact, of the 97 references in the TTASAAN report, only 12 are from 1993 or later. Furthermore, the early gamma cameras used for SPECT neuroimaging were single-headed cameras with limited resolution. Thus, the conclusions provided in the TTASAAN report were premature.

In the 25 years since the TTASAAN report, the American Academy of Neurology (AAN) has never deemed to re-examine their premature position on the use of brain perfusion SPECT in the evaluation of traumatic brain injury (TBI), stroke, seizure disorders, dementia, and neuropsychiatric conditions. Despite extensive advances in technology, software, and technique, as well as, for example, the publication of over 120 research studies and articles and published data from over 23,000 subjects on the use of SPECT just in the evaluation of TBI, the AAN has largely taken an overcautious, and sometimes dismissive, position toward the use of SPECT scans. In contrast, the European Association of Nuclear Medicine (EANM) (2) has deemed that brain perfusion SPECT scans are appropriate for the evaluation of TBI and that SPECT scans have predictive value in the clinical outcome of TBI (2). Moreover, the Canadian Association of Nuclear Medicine recently has issued guidelines on the use of SPECT imaging in the evaluation of TBI, stroke, dementia, neurotoxicity, and psychiatric conditions (3).

Curiously, the authors of the TTASAAN report made it clear that this report was neither a position paper nor a solidified assessment of SPECT. The authors unambiguously articulated that the assessment was to be revised as the field advanced. Quoting from the opening paragraph of the TTASAAN report (1):

*“This paper, provided to the Academy membership as an educational tool, will be subjected to periodic revision as new information becomes available.”*

The periodic revision has never occurred. In many ways, this is the equivalent of assessing Xray computed tomography (CT) in its infancy and then never re-assessing its merit thereafter. The first CT scanner became commercially available in 1972. CT neuroimaging was met with skepticism among neurologists (4). CT neuroimaging was deemed, “a passing fancy.” The American Neurological Association published a report in 1975 stating the new method of imaging the nervous system would greatly reduce the need for neurologists. Numerous articles in the late 1970's criticized CT neuroimaging for its failure to accurately detect brain pathology (5–8) during the early years of its clinical use. In 1975, a commission of the AAN reported

*“A CT scan can give a more accurate localization in far less time than a neurologist. Ultrasound of the carotid arteries can localize and indicate the degree of stenosis more accurately than a clinician with a stethoscope. The portent for the future is that neurologists who rely exclusively on their wits and their pins and hammers, unaware the machine age has finally come to neurology, may become obsolete”* (9).

Despite the initial skepticism and concern that CT neuroimaging would replace the need for neurologists, the use of CT scanning expanded exponentially. CT neuroimaging is now a cornerstone of neurological evaluations, particularly in the acute setting. Nonetheless, as will be elaborated below, CT is very limited in what it can reveal about brain function.

In the same 25-year period, functional magnetic resonance imaging (fMRI) has undergone explosive growth. Hundreds of millions of dollars in research funding in the United States have been poured into fMRI studies. According to PubMed, over 40,000 research articles have been published. Despite the massive investment of time and effort, fMRI has yet to provide a clinically useful diagnostic tool for assessing brain function in individual cases. Indeed, the American Psychiatric Association recently issued a position paper (10) stating

*“(fMRI) neuroimaging has yet to have a significant impact on the diagnosis or treatment of individual patients in clinical settings.”*

Moreover, a recent analysis of post-processing statistical validity revealed that a potentially staggering 70% of fMRI studies had false positive results (11), which could mean much of the fMRI research findings are invalid. The AAN has seemingly scrupulously ignored this serious caveat to the application of fMRI in research or clinical practice (12).

Similarly, extensive funding and effort has been invested in diffusion tensor imaging. However, it has been plagued by inconsistencies across centers due to technical elements arising from different hardware, competing software, varying sequences, dissimilar reconstruction algorithms, and technique. For example, eddy current distortion is often found to be larger than the acquisition voxel size (13). While a thorough analysis of diffusion tensor imaging is beyond the scope of this review, it will suffice to say that anisotropy can be either elevated or depressed following TBI. There have been numerous studies with contradictory findings (14, 15). Variations in technique or the unreliability of diffusion tensor imaging in TBI has been suggested as the cause for the conflicting data (14, 15).

Thus, while the AAN, and neurologists in general, have distanced themselves from SPECT neuroimaging based, in part, on the now outdated TTASAAN report and embraced other technologies, they have been left with technically flawed methods of visualizing brain function (11, 14, 15). Meanwhile, extensive advancements have been made in the practice and technology of perfusion SPECT neuroimaging and massive databases have been accumulated. Together, these factors lead to the need to re-examine the policy and practice set forth by the AAN in 1996.

### Defining SPECT

SPECT is a type of nuclear medicine scan to create 2-dimensional (2-D) and 3-dimensional (3-D) pictures of functional processes within the patient's body. A radiopharmaceutical is administered to detect specific activities within the body. A gamma camera measures the radiation emitted by the radiopharmaceutical and rotates around the patient to acquire a set of 2-D planar images. Using a reconstruction technique, these planar images are reconstructed into a 3-D volume from which slices at various angles can be extracted to visualize the distribution of activity within the patient's body. In the case of perfusion SPECT neuroimaging, the radiopharmaceutical is transported via the bloodstream and is quickly taken up by neurons (16) (see below), such that the uptake of radiotracer is dependent upon, and therefore reflective of, the regional cerebral blood flow (rCBF). Cerebral blood flow at the level of cortical columns or functional subregions is regulated by neuronal activity. Increased activity induces increased local blood flow, while decreased activity results in reduced blood flow. The detection of the radiotracer uptake throughout the brain allows the clinician to identify both areas of hypoperfusion (hypo-functioning) and of increased perfusion (hyper-functioning). SPECT post-processing generates tomograms and a 3-D mapped representation of the brain, *ideally* with color-coded intensities proportional to rCBF which correlate with the brain function in that region.

## Assessment of Guidelines and Implications

### The Technical Aspects of Early SPECT Neuroimaging

Brain perfusion SPECT scans were initially developed in the 1970's. After the development of the Anger scintillation detector in 1950 (17) and the invention of the Anger circuitry in 1969 (17), several groups developed scintillation cameras. Paul Harper et al. with the University of Chicago first explored transaxial tomography using the Anger camera (18). The first whole body SPECT cameras were developed by 1976 (17). Brill et al. at Vanderbilt University and Jaszczak et al. at Searle Radiographics independently developed brain-specific gamma cameras.

While the technology of the scintillation camera was ongoing, the development of brain specific tracers was advancing rapidly. The early SPECT cerebral blood flow studies utilized ^133^Xenon which can provide a quantitative measure of cerebral blood flow. However, ^133^Xenon has relatively low energy and required a special breathing apparatus which was cumbersome and uncomfortable for the patient. Tracers with high energy gamma radiation were sought and ^123^Iodine (^123^I) or ^99m^Technetium (^99m^Tc) became the primary candidate radiolabels. An ^123^I tracer, ^123^I-N-isopropyl-iodo-amphetamine (^123^I-IMP), was developed in 1980 for cerebral perfusion using iodinated amphetamine. It could be tagged with either ^123^I or ^125^I. This tracer showed high brain extraction and linear uptake over a wide range of blood flow rates (19). It remains in use today; however, there were some distinct disadvantages to this tracer. The first is the use of ^123^I requires pretreatment to protect the thyroid. The second is that the tracer is redistributed from the lungs to the brain over the first 20 min after injection leading to a smearing of activity over time. Brain areas demonstrating high cerebral blood flow at the time of injection may not maintain that level of blood flow during the 20-min interval when ^123^I-IMP is cleared from the lungs and accumulates in the brain (16).

The more lipophilic agent propylene amine oxime, which could be labeled with ^99m^Tc, was explored as a brain perfusion tracer during the late 1970's and early 1980's. Initial agents had rapid clearing and so required fast imaging. In 1985, a methylated version of the agent was developed, which had almost ideal properties—^99m^Tc- hexamethylpropyleneamine oxime (HMPAO) (20). First, ^99m^Tc has a long half-life of 6 h and can be made independent of a cyclotron. Second, neither ^99m^Tc nor HMPAO interfere with any biological processes in the body, unlike ^123^I or the amphetamine tracer. Third, HMPAO is retained in the cell due to conversion to a less lipophilic form, which reduces washout and prolongs the window for scanning to several hours (16, 20–22). Fourth, the accumulation of HMPAO in the brain is very rapid, being virtually complete in 40 s and it maintains a fixed distribution after 5 min (16, 22). Thus, the scan measures the cerebral blood flow at the time of injection, not at the time the scan is actually performed. In essence, a SPECT scan captures a frozen image of brain function at the time of injection (16, 22). This makes ^99m^Tc-HMPAO ideal for capturing brain activity during transient conditions (e.g., seizures, transient ischaemic attacks) or psychological challenges (e.g., concentration tasks). At very high perfusion rates, ^99m^Tc-HMPAO accumulation and back diffusion becomes disproportional; thus, ^99m^Tc-HMPAO uptake is non-linear compared to ^133^Xenon (16, 23). Lassen et al. (23) proposed a correction algorithm which has been shown to closely approximate rCBF in comparison to ^15^CO_2_ PET (24). Nevertheless, accumulation is linear within the normal physiological range in the human brain (16, 23).

### Limited Stability of HMPAO Pre-1996

At the time that ^99m^Tc-HMPAO was originally approved by the FDA in 1988, a key technical flaw still remained. The agent was unstable and would decompose rapidly after reconstitution. Realistically, it was only viable for 30 min after reconstitution (16). The work of quality checking, measuring the radioactivity, calculating and drawing up the dose, and patient preparation had to be accomplished very hastily prior to injecting the patient. As a result, the quality of the SPECT images varied greatly based on the deftness and technique of the technologist handling the tracer. In 1993, the addition of methylene blue proved effective in stabilizing HMPAO for several hours after reconstitution. This contributed to improved scan quality.

**Figure 1:**
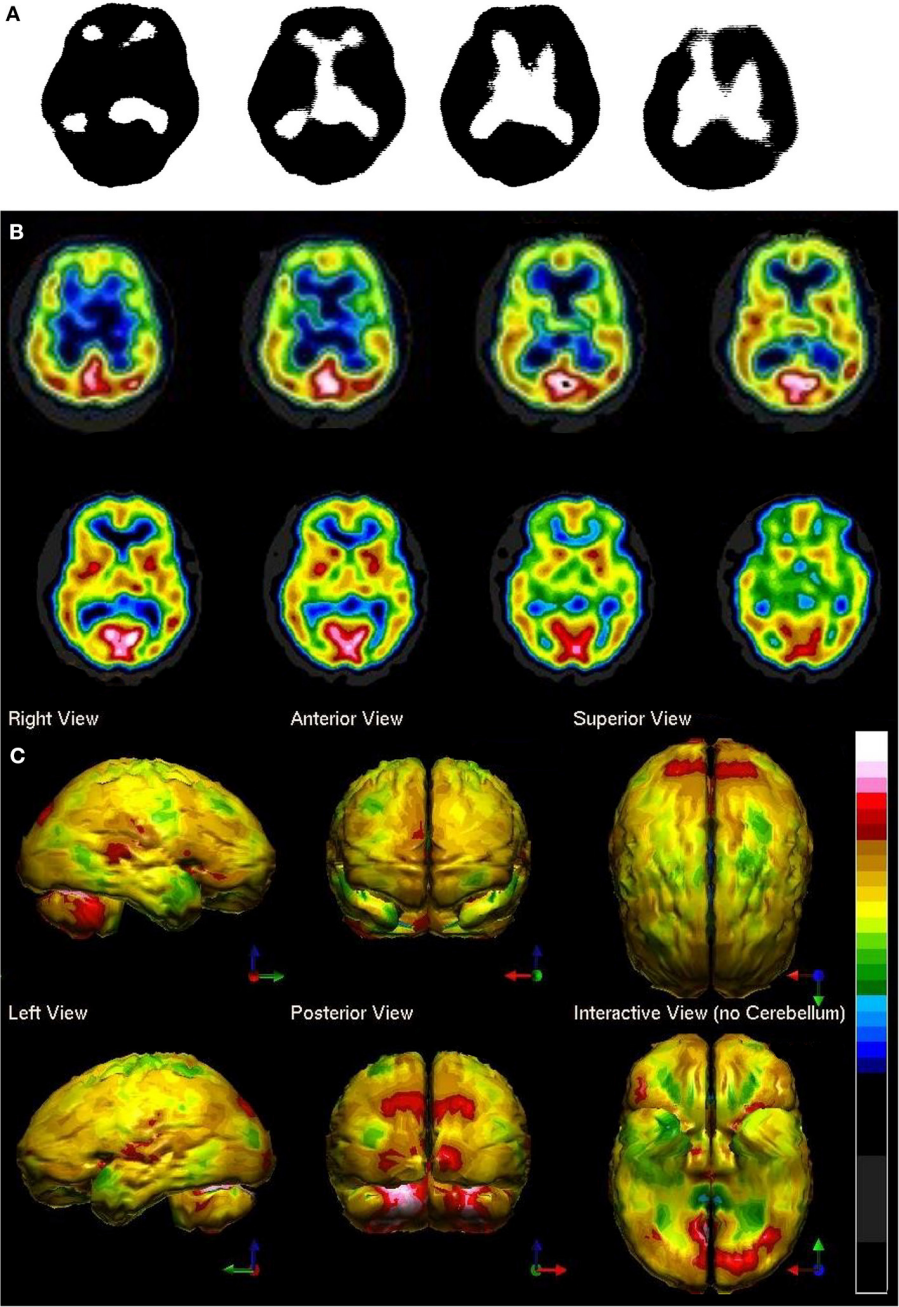
Perfusion SPECT scan of normal control. **(A)** Four horizontal tomograms from a ^99m^Tc-ECD perfusion SPECT scan performed on a single-headed gamma camera in 1989 [The figure was originally published in JNM. © SNMMI (25)]. **(B)** Eight horizontal tomograms from a modern ^99m^Tc-HMPAO perfusion SPECT scan obtained from a dual-headed gamma camera. The color scale is scaled relative to the patient's mean cerebral perfusion. Mean blood flow (72%) is in yellow. Color shifts occur at approximately every 0.5 SD (3%) relative to the patient's mean. Details of the brain can be appreciated, including the thalamus, head of the caudate nuclei, lentiform nuclei, anterior cingulate gyri, and distinct cortical regions. **(C)** The modern SPECT scan displayed in 3-dimensional reconstruction. Increased perfusion in the visual cortex and the slightly lower average perfusion level in the temporal lobes bilaterally can be appreciated. The color scale is the same as **(B)**.

HMPAO was not truly stabilized for clinical application until 1993. In addition, most of the brain SPECT perfusion studies pre-1994 were performed on single-head gamma cameras. Since, then the quality of SPECT neuroimaging has greatly improved with the use of multi-head gamma cameras (See Figure 1). In addition, refinements in post-processing, as well as the introduction of statistical comparison to normative databases, have greatly enhanced the quality and diagnostic capacity of SPECT scans.

### Display Limitations

Early SPECT studies were compromised by limitations of post-processing and display. Methods for correcting energy attenuation were imprecise. Displays were often essentially binomial—-above a certain threshold (1) the display showed black and below that threshold (0) the display showed an absence of black, as illustrated in Figures 1A, **3C**. Some effort at a gray scale was introduced by Ismael Mena and his group at Harbor UCLA as illustrated in **Figure 3B**. The challenge with SPECT scans is that they are detecting changes in *degree* of function, which is displayed as changes in the *intensity* of the signal. Greyscale permits finer details to be seen, making it ideal for anatomical MRI; however, when discerning changes in intensity over large areas, color displays improve detection. For example, Stapleton et al. (29) examined this issue using SPECT scan data. The study involved the use of scan data from one-half of a brain, which was then inverted to create a symmetrical template of a brain. Then an artificial defect was created in the cerebellum by decreasing pixel values in the designated area by 1–12.5%. This construct was displayed in greyscale, a red color scale similar to “heated object,” and blue/green/red where low counts were blue, mid counts were green and high counts were red. Despite the expressed bias toward greyscale among the radiologists tested, subject readers detected the artificial lesion much better in either of the color scales. In fact, the more subtle the lesion (pixel value decreases < 10%), the better color aided in detecting the lesion.

Humans, like all primates, have superior discrimination of color vision (30). One need only look at a Monet painting in greyscale to see the importance of color is discerning complex visual information. While greyscale allows superior detail discrimination, it does not foster the detection of changes in intensity. This was more recently demonstrated in fluid-attenuated inversion recovery (FLAIR) anatomical MRI in stroke (31). A large retrospective sample of FLAIR images were displayed in greyscale and a color scale. The addition of color increased detection of stroke and inter-rater agreement by 23%. The positive predictive value similarly increased from 85.3 to 95.7% (31).

**Figure 2:**
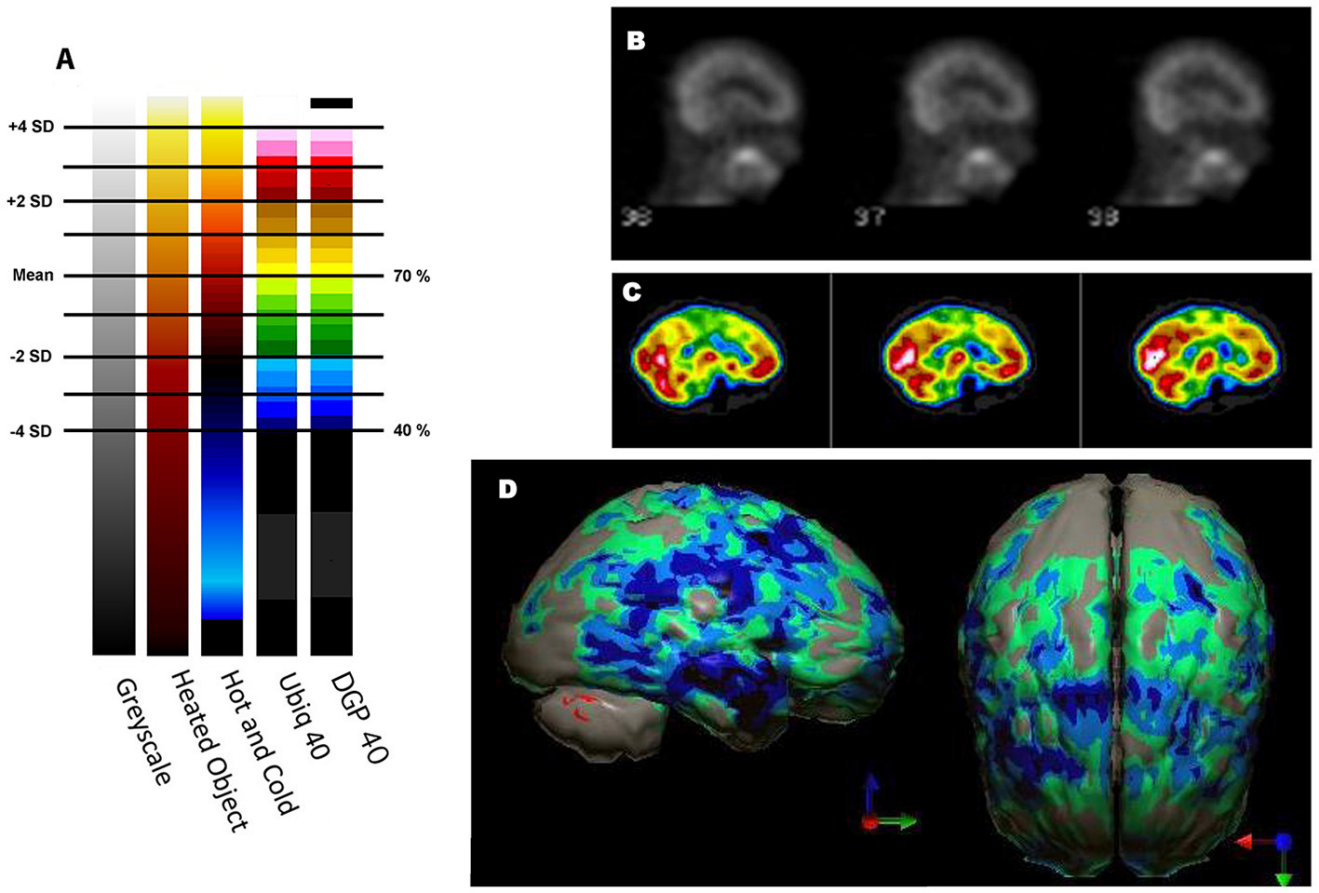
Humans, like all primates, have superior discrimination of color vision. While greyscale allows superior detail discrimination, it does not foster the detection of changes in intensity. Since functional neuroimaging is about changes in the intensity of the signal, it is important that the observer can readily detect small changes. **(A)** Various commonly used color scales and a greyscale are displayed. The mean cerebral perfusion in the human brain is 70.3% of the maximal flow with a standard deviation (SD) of 8.35%. The mean and ± 1, 2 SD, 3 SD, and 4 SD are indicated. A change of ± 2 SD is unlikely to be appreciated in greyscale but can be readily distinguished in Heated Object, Ubiq40, and DGP40 color scales. An increase of 2 SD can be distinguished in Hot and Cold color scale, but a decrease of 2 SD or less would not be discernable. A 1 SD increase or decrease would be difficult to discern in greyscale, Heated Object and Hot and Cold color scale, but are readily detected in Ubiq40 and DGP40. **(B)** A representative low quality ^99m^Tc-HMPAO perfusion SPECT scan demonstrates poor technique with the inclusion of extracranial structures. The scan was read in greyscale and interpreted as a normal scan. **(C)** The same patient was rescanned with proper technique. Decreased perfusion in the posterior frontal and temporal cortices can be appreciated when viewed using the Ubiq40 color scale. **(D)** The patient's data is compared to a normative database (*N* = 68). A map of statistically significant differences can be generated using the Oasis software by Segami, Inc. Here, the color scale indicates gray for areas that do not differ significantly from the normative database. In contrast, areas of green, light blue, and dark blue represent areas of more than 2, 3, and 4 SD below the mean perfusion of the normative database, respectively. Statistically significant increases in perfusion are illustrated in the red color scale. Decreased perfusion in the bilateral temporal cortex and bilateral posterior frontal cortex, but with sparing of the anterior cingulate gyri, can be appreciated. The findings are consistent with mild cognitive impairment of the frontal-temporal variant and the patient showed consistent findings on neuropsychological assessment.

Ismael Mena et al. at Harbor UCLA did extensive studies with normal subjects with ^133^Xenon to determine quantitative data on the normal range of cerebral blood flow in humans (32). In a brief summary of an extensive body of work, mean cerebral blood flow was determined to be 70.3% of the maximum cerebral blood flow. The standard deviation (SD) was 8.35%. This work has been corroborated by several others and summarized by Devous et al. (33). Figure 2 illustrates the mean ± 1 and ± 2 SD set against various color scales and greyscale. One can quickly see that in greyscale neither an increase in 2 SD nor a decrease in 2 SD can be detected. In contrast, in the Heated Object color scale a decrease of 2 SD can be easily discerned. It is less clear that a decrease of 1 SD or an increase of 1 or 2 SD could be detected in Heated Object scale. In the Hot and Cold color scale, both increases and decreases of 2 SD could be easily detected, but changes of 1 SD might be more challenging. Lastly, the Ubiq 40 color scale, developed by Ismael Mena based on his quantitative data, and the DGP40, developed by one of the authors - DGP, has incremental color changes at approximately 2.7%. Changes in perfusion as small as 0.3 SD can be detected in either direction. The distinction between the DGP40 and the Ubiq40 is that the top 2% of the DGP40 is black, which allow easy identification of the most active or highly perfused part of the brain. An illustrative case is provided in Figure 2—a patient with signs of mild cognitive impairment and decreased performance on neurocognitive testing. The scan in greyscale is read as normal (Figure 2B). The tomograms in Ubiq40 show subtle decreases in perfusion in the frontal and parietal cortices (Figure 2C). However, when the scan is compared to a normal database of age-matched controls, statistical analysis reveals a pattern of hypoperfusion consistent with mild cognitive impairment of the frontal-temporal variant. This case illustrates the value of using color scale for SPECT scans wherein changes in intensity are more important than anatomical detail.

Throughout this article, early SPECT scans in greyscale will be contrasted with modern SPECT scans using color display and statistical comparison to a normal database. The wealth of information that becomes visible in color scale is self-evident. Nonetheless, despite extensive research supporting the value of color display, many radiologists and nuclear medicine physicians persist in using greyscale to read SPECT scans.

## Assessment of the State of the Art—Spect in Brain Disorders

Herein, we will provide technical background on the studies cited by the TTASAAN report and then provide technical details from state-of-the-art studies in modern brain perfusion SPECT imaging. We will begin with and illustrate most extensively the state of the art in the evaluation of head trauma and traumatic brain injury (TBI), because this indication has been quite controversial and has raised the most strident criticisms.

### Head Trauma

The definition of TBI has evolved since the publication of the TTASAAN report. At that time, “concussion” was considered a transient state. Now concussion is recognized as a form of TBI, despite an absence of a loss of consciousness (LOC). Because of this shift and the recognition that: 1) concussions can have persistent effects on the brain (34, 35), 2) repeated concussions can have cumulative damage (36), and 3) persistent pathological changes can occur following even a single concussive event (37, 38), we refer to the Centers for Disease Control (36) for a basic definition of the levels of severity of TBI.

The World Health Organization defines post-concussion syndrome (PCS) as “persistence of a constellation of physical, cognitive, emotional and sleep symptoms beyond the usual recovery period after a concussion” (39), including 3 or more of the following after head injury: headache, dizziness, fatigue, irritability, insomnia, reduced tolerance of stress, concentration difficulty, or memory difficulty.

**Figure 3:**
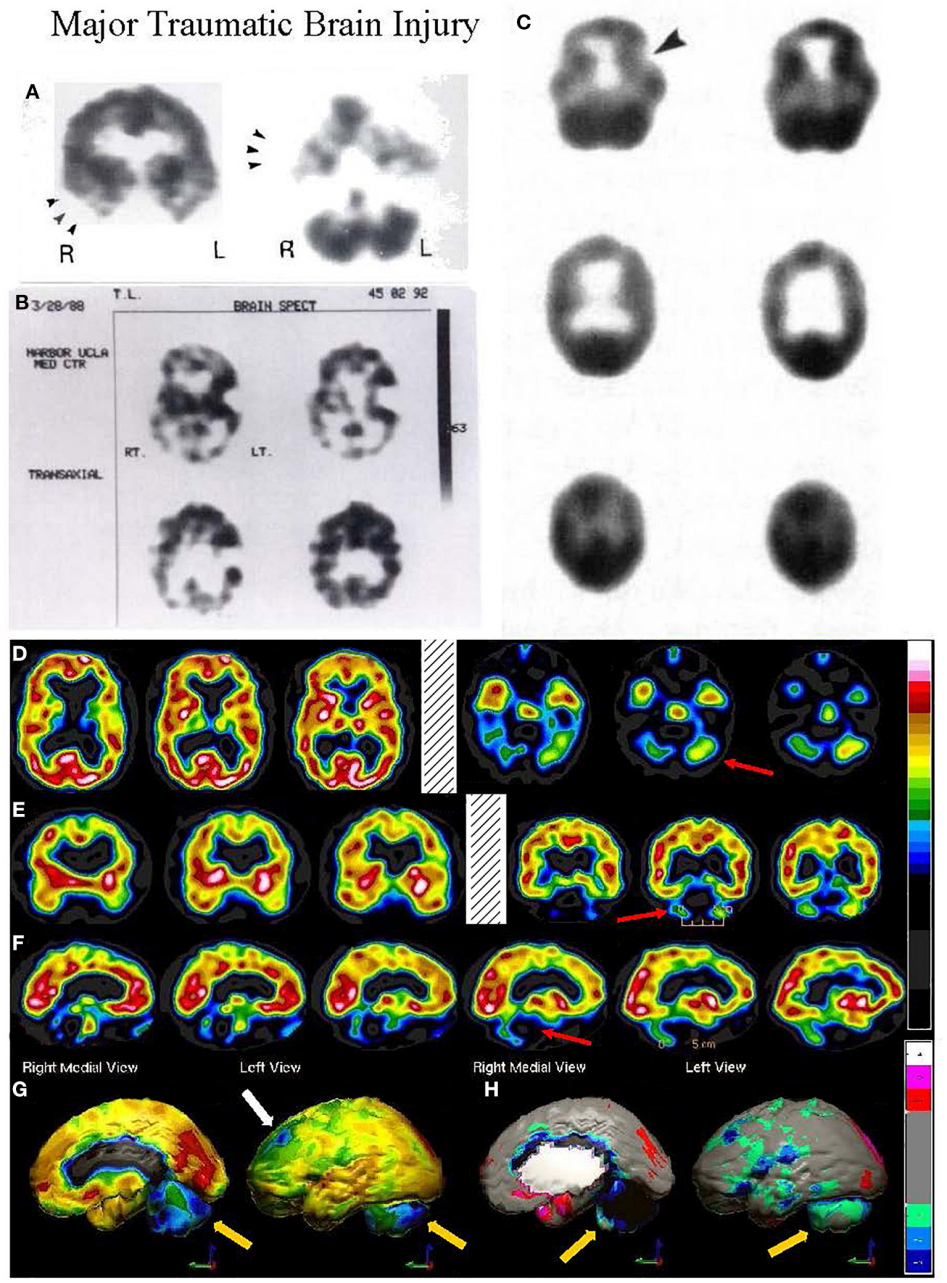
**(A–C)** Examples of SPECT scans cited in TTASAAN report. Anatomical details are lacking. **(A)** 40-year-old female with major head trauma showed decreased perfusion of the right temporal lobe, whilst CT and MRI scans were normal [The figure was originally published in JNM. © SNMMI (26)], **(B)** 45-year-old male thrown from a horse showed decreased bilateral occipital perfusion [The figure was originally published in JNM. © SNMMI (27)]; **(C)** 37-year-old female with major head trauma from a motor vehicle accident (MVA). Perfusion is decreased in the bilateral frontal and temporal lobes [The figure was originally published in JNM. © SNMMI (28)]. **(D–F)** A 19-year-old woman was involved in a head-on collision MVA as a passenger. She suffered severe trauma to the back of her head. A modern ^99m^mTc-HMPAO perfusion SPECT scan was performed with a dual-head camera. **(D)** Horizontal tomograms (non-sequential, break in sequence shown by cross-hatched bar) illustrate intact cortical function but marked hypoperfusion in the cerebellum bilaterally (red arrows). **(E)** Coronal tomograms (non-sequential). **(F)** Sagittal tomograms (sequential). **(G)** 3-D representation of SPECT scan data illustrating a small area of marked hypoperfusion in the left frontal cortex (white arrow) and profound hypoperfusion in the cerebellum (yellow arrows) which is more pronounced in the medial aspects. **(H)** The patient's data is compared to a normative database using Segami Inc. Oasis software. The color scale is the same as in Figure 2D. The injury to the left frontal cortex and lateral aspects of the frontal cortex can more clearly be visualized. Area of white in the right medial view is an area where there is no statistical comparison data.

**Figure 4:**
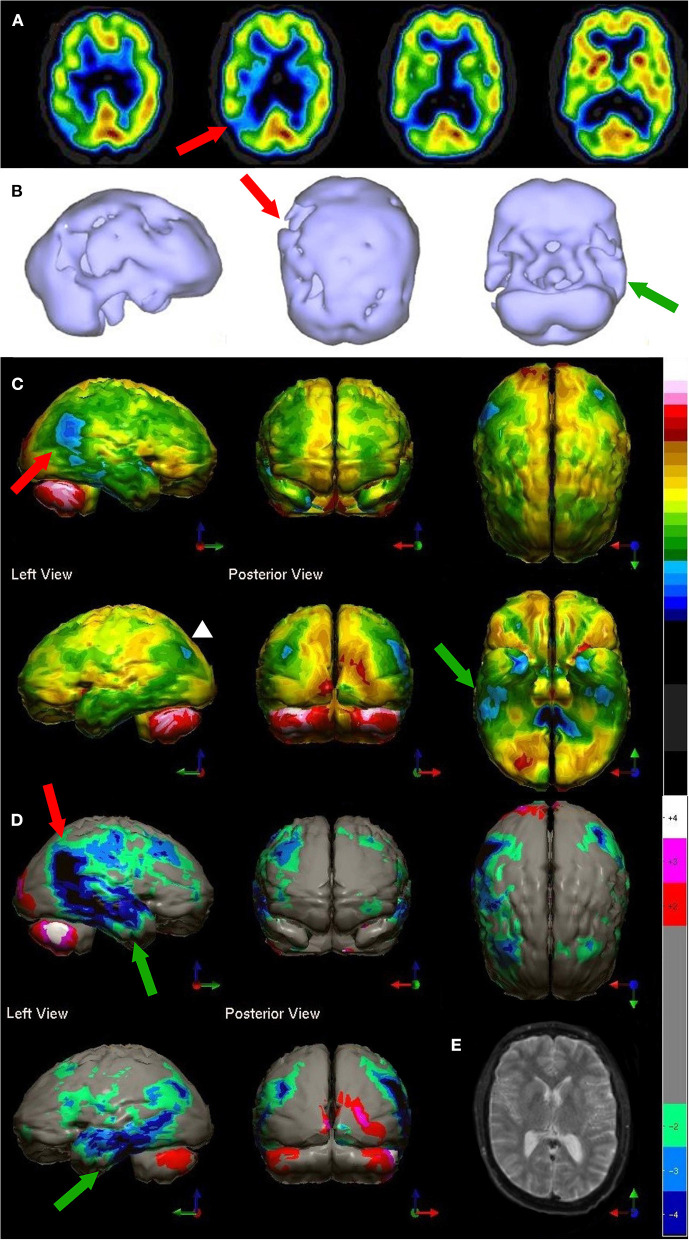
Tomographic and multiple 3-D representations of major TBI. A 58-yr-old female was struck on the right parietal region by a heavy object with loss of consciousness of approximately 2 hours. Perfusion SPECT scan was performed seven years after the injury with ^99m^mTc-HMPAO and a dual-head gamma camera. **(A)** 4mm horizontal sections illustrate decreased perfusion in the right parietal region (red arrow). The color scale is the same as Figure 1B. **(B)** SPECT data can be displayed in 3-D representations that facilitate the identification of large, diffuse, or subtle lesions. Here, data is presented as an isocontour display wherein cortical areas which fall below 60% of the maximal cerebral blood flow are displayed as a depression or hole. The large parietal defect is apparent on the right (red arrow), as well as bilateral temporal lobe hypoperfusion (green arrow). **(C)** Another 3-D representation utilizes the same color scale as **(A)**. The right parietal defect appears as an area of blue and green (red arrow). A contra-coup injury can be visualized in this representation (white arrowhead). Temporal lobe hypoperfusion is again evident bilaterally (green arrow). **(D)** The patient's data is compared to a normative database using Segami Inc. Oasis software. The color scale is the same as in Figure 2D. The parietal lobe injury (red arrow) and the contra-coup injury are easily visualized, along with more diffuse penumbra injury and bilateral lateral temporal lobe hypoperfusion (green arrows). **(E)** Anatomical MRI completed at the time of the SPECT scan showed no abnormalities. Section at same level as far right horizontal tomogram in **(A)**.

**Figure 5:**
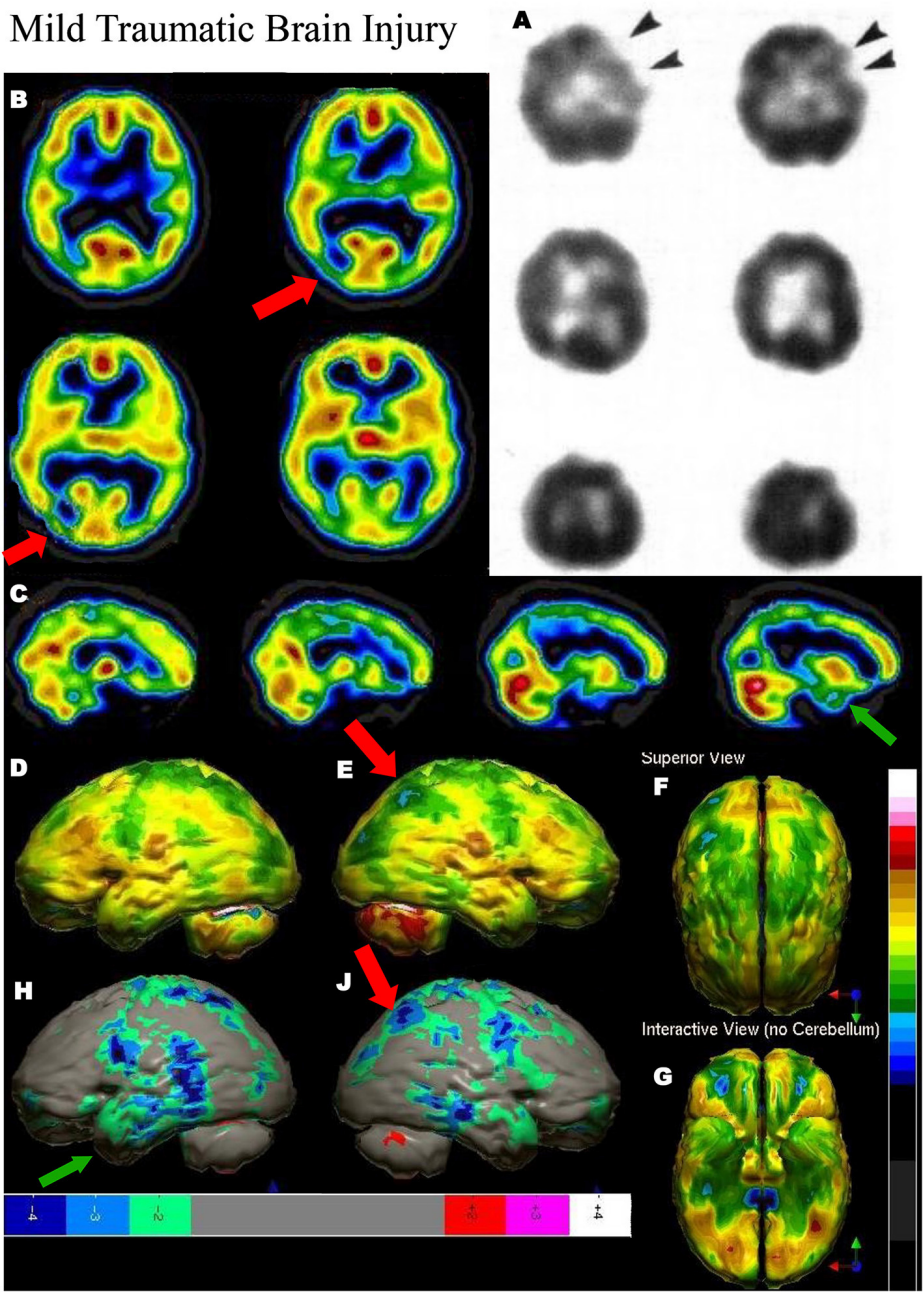
Mild TBI. **(A)** Example of SPECT scans cited in TTASAAN report. Anatomical details are lacking. 50-year-old male with minor head trauma after a motor vehicle accident showed decreased perfusion of the left inferior frontal and left anterior temporal lobe, whilst MRI scan was normal [The figure was originally published in JNM. © SNMMI (28)]. **(B–G)** A 2019 SPECT scan using a dual-headed gamma camera of an 18-year-old male who struck a tree while mountain biking and briefly lost consciousness. A modern ^99m^mTc-HMPAO perfusion SPECT scan was performed with a dual-head gamma camera. **(B)** Horizontal tomograms show detail of thalamus, anterior cingulate, caudate, and lentiform nuclei. A focal area of hypoperfusion can be seen in the right parietal cortex (red arrow). Color scale is the same as in Figure 1B. **(C)** Sagittal tomograms reveal decreased inferior frontal perfusion and decreased medial temporal perfusion bilaterally (green arrow). **(D–G)** 3-D representation of SPECT scan data showing left lateral **(D)**, right lateral showing the area of hypoperfusion in the right parietal cortex (red arrow) **(E)**, superior **(F)**, and inferior views **(G)**. **(H–J)** The patient's data is compared to a normative database using Segami Inc. Oasis software. The color scale is the same as in Figure 2D. Areas of relatively decreased perfusion are more evident, such as the area of hypoperfusion in the right parietal cortex (red arrow).

The TTASAAN report (1) included seven early studies of TBI with a total of 253 subjects. All studies were conducted on single-head gamma cameras. Two studies utilized ^125^I-IMP and five studies utilized ^99m^Tc-HMPAO with three of those studies conducted prior to the stabilization of HMPAO. All scans were assessed visually only. Jacobs et al. (40) will be discussed in detail below. Abdel-Dayem et al. (41) examined a series of 14 acute moderate-to-severe TBI cases with HMPAO SPECT scans performed within 72 h. Seven of the 14 cases did not survive. The number and extent of lesions observed by SPECT were compared to the number and extent of lesions seen by CT scan. Ducours et al. (42) examined 10 comatose TBI patients and 10 patients with TBI, but no LOC. All had a negative CT scan and a ^125^I-IMP perfusion SPECT scan. Patients without LOC had a normal ^125^I-IMP scan, while 9 out of 10 of the comatose patients had functional deficits on SPECT scan. Roper et al. (27) examined CT and HMPAO perfusion SPECT scans in 15 patients with mild, moderate or severe head injury (Figure 3B). SPECT revealed more focal lesions than CT. Gray et al. (28) examined 53 chronic (>6 months) TBI patients (20 mild, 33 severe TBI) compared to 14 normal controls using HMPAO SPECT (Figure 3C) and comparison to CT. Over 90% of the chronic severe TBI cases had areas of decreased perfusion on SPECT, but only 72% showed abnormalities on CT scan. Conversely, 100% of the patients with a normal SPECT scan had a normal CT scan. Ichise et al. (26) examined 29 chronic (> 6 months) TBI patients (15 mild, 14 severe TBI) and compared the HMPAO perfusion SPECT scans and neuropsychological testing results to those of 17 normal controls (Figure 3A). Trail Making A and B, Digit Symbol, and Wisconsin Card Sorting stood out as tests which strongly differentiated brain injured patients from controls (*p* < 0.001). Most lesions were in the frontal and temporal lobes and correlated with decreased neuropsychological scores on memory, attention, and executive function (26). Masdeu et al. (43) attempted to utilize negative controls (normal control) and positive controls (human immunodeficiency virus encephalopathy {HIV}) to examine mild TBI. Fourteen patients with mild TBI underwent CT scan and IMP or HMPAO perfusion SPECT scans within 48 h of head trauma. The results were compared to 15 normal controls and 12 patients with HIV encephalopathy. None of the normal controls were read as TBI; however, 40–50% of the TBI cases were read as HIV encephalopathy and 14–28% of the TBI cases were read as normal. The latter study highlights the jeopardy involved in visually interpreting SPECT scans, particularly in greyscale. As detailed above, the human eye is unable to separate 2 standard deviations in greyscale, because it is designed for color vision. Areas of hypoperfusion of <2 SD will be missed by visual read. Moreover, the absence of statistical comparison to a normative database or a matched set of normal also risks false negatives. This is strikingly demonstrated in Figures 3–5.

We (TAH, DGP), along with our colleagues, published a systematic review in 2014 which examined the entire extant literature on SPECT scans in the evaluation of TBI (44). The systematic review showed Level IIA evidence (at least one randomized controlled trial) for the utility of brain SPECT in TBI. The review identified 52 cross-sectional studies and 19 longitudinal studies including a total of 2,634 individuals over 30 years of literature. In addition, seven studies which were not included in the systematic review contain 223 subjects (45–51). Subsequently, two large retrospective studies comparing SPECT neuroimaging in TBI, post-traumatic stress disorder (PTSD), and normals containing over 21,399 subjects have been published (52, 53). Thus, besides the studies included in the TTASAAN report (1), there are an additional 69 studies containing 23,944 subjects on the utility of perfusion SPECT neuroimaging in the evaluation of TBI.

In addition, there have been numerous editorials and opinion pieces criticizing the use of SPECT to evaluate TBI (54–56). Central to the criticism is there is no gold-standard SPECT finding for TBI, particularly mild TBI. Another criticism is that many of the studies of TBI have a small number of subjects and lack a control group (54–56). A third criticism is SPECT may not provide additional benefit over CT or MRI (55, 56). A fourth criticism is that SPECT findings do not correlate with neuropsychological testing (54–56). A fifth criticism is that it has been unclear that SPECT scans can predict clinical outcome (54–56). Each of these criticisms will be addressed in turn.

#### Lack of Gold-Standard Finding

This position reflects an almost foolish belief that such a gold-standard could or should exist. By its very nature, TBI is highly variable. The mechanism of injury (impact, rotational, etc.), point of impact, presence or absence of contra coup injury, handedness, presence of prior injury, and other factors all contribute to the manifestation of TBI in each patient (34, 38, 57). The neuropsychological sequalae of injury will also depend upon what areas of the brain are affected, the inter-connectedness of affected areas, handedness, nutrition, toxic exposures, premorbid intelligence, and history of prior injury (58). This confound is not limited to SPECT, but also plagues other forms of neuroimaging, such as diffusion tensor imaging, when applied to the evaluation of TBI.

#### Small N Studies or Lack of a Control Group

By the very nature of TBI, it is not possible to have a randomized study of neuroimaging applied to TBI. How does one recruit a sample of subjects with normal baseline SPECT scans and then subject them randomly to a head injury followed by a repeat SPECT scan? As a result, there can never be true Class I evidence (well-designed, randomized, controlled clinical trial), as defined in the TTASAAN report (1), for the diagnostic and/or prognostic effectiveness of SPECT in the situation of TBI. This same limitation applies to studies of fMRI, CT, anatomical MRI, MEG, and diffusion tensor imaging. An early approach to this barrier was to randomly present cases of TBI with positive and negative controls to reading physicians (43). The study was technically flawed as described above.

Of the 54 published cross-sectional studies of perfusion SPECT neuroimaging in the evaluation of TBI, 26 had 20 subjects or less. Seven studies had 100 subjects or more (52, 53, 59–63). One study had over 7,600 subjects with TBI (53). Of the 19 longitudinal studies, nine had 20 subjects or less. Four longitudinal studies had 100 subjects or more (64–67). For example, Gowda et al. (66) prospectively performed CT and perfusion SPECT scans on 92 patients with acute TBI. Both scans were performed within 72 h of injury. Abnormal SPECT scans were found in 63% of cases—-half of these cases had normal CT scans. Two patients showed CT abnormalities without corresponding SPECT findings. A subarachnoid hemorrhage was the finding in both cases. The Newcastle-Ottawa Scale (NOS) was developed to assess the quality of non-randomized studies (68). The scale was applied to all the longitudinal SPECT studies by Raji et al. (44). The mean score for the 19 studies was 6 ± 1.4, which is considered to be high quality (NOS range 0–9).

Among the 71 studies included in the aforementioned systematic review (44), 15 included a control group. In addition, Stamatakis et al. examined SPECT scans and MRI from 51 subjects with TBI using statistical parametric mapping in comparison to 32 subjects in a control group (47). Atighechi et al. examined 21 subjects with TBI and anosmia compared to positive and negative control groups (50). Amen et al. conducted a retrospective comparison of TBI and PTSD (52). All patients underwent extensive psychiatric interview and completion of a battery of questionnaires. The diagnosis was made by Board-certified psychiatrists based on DSM-IV or V criteria. Baseline perfusion SPECT scans differentiated TBI from PTSD with a sensitivity of 92% and a specificity of 85% (52). Amen et al. replicated these findings in a separate retrospective evaluation of SPECT scans from distinct and closely matched groups of patients with TBI (*N* = 104), PTSD (*N* = 104), both TBI and PTSD (*N* = 73) and 116 healthy controls (53). All patients were diagnosed by a similar extensive battery of questionnaires and psychiatric interview. Controls were found to be free of psychiatric conditions, TBI, or substance abuse by extensive psychiatric interview and completion of a battery of questionnaires using DSM-IV or V criteria. The baseline perfusion SPECT scans were compared visually and by quantitative region of interest analysis. TBI could be distinguished from controls with a sensitivity of 100% and a specificity of 100% in both visual reads and quantitative analysis. In distinguishing TBI from PTSD, the sensitivity was 100% and the specificity was 100% for quantitative analysis and a sensitivity of 86% and specificity of 81% for visual reads. In addition, they conducted a larger comparison of 7,505 patients with TBI and other psychiatric comorbidities compared to 11,147 psychiatric patients without TBI who served as controls (53). With this more diverse group, sensitivity was 70% and specificity was 54% for both visual reads and quantitative analysis. The comparison of TBI and PTSD yielded somewhat higher accuracy with a sensitivity of 80% and a specificity of 60–62% (53).

In summary, there are numerous large-N cross-sectional, longitudinal and retrospective studies of the utility of SPECT in the evaluation of TBI. Indeed, thousands of subjects have been compared to hundreds of controls across 18 studies.

#### Does SPECT Provide Additional Information Over CT or Anatomical MRI?

Since perfusion in the gray matter is regulated by neuronal activity, as described above, perfusion SPECT provides a method of detecting neuronal dysfunction in the absence of anatomical change. Areas of the brain which are stunned, surviving, but not functioning (as in the ischemic, or otherwise functionally compromised) show no anatomical changes. However, the decreased function can lead to decreased perfusion. We (TAH) have demonstrated this in a case of chronic TBI wherein cerebral perfusion surrounding the injury and even in the contralateral hemisphere was decreased, despite normal appearance of the involved areas on MRI (69). These affected areas responded to treatment and showed improved perfusion upon repeat SPECT imaging. Acute TBI (within 72 h) represents a unique situation wherein perfusion can increase or decrease depending upon a number of factors, such as neuronal dysfunction and shutdown, inflammation, changes in blood-brain barrier permeability, excitotoxicity, and more. For example, Obrist et al. (70) performed serial quantitative perfusion SPECT (^133^Xenon) scans and found that rCBF was initially reduced (12 h) and then increased to hyperemic levels at 57 h after injury. Hyperemia was associated with increased intracranial pressure. The changes in perfusion may be due to loss of autoregulation (71) and/or transient disruption of the blood-brain barrier (72).

The collective literature (44) indicates that perfusion SPECT scans are superior to CT scans for detecting functional injury following head trauma in subacute and chronic TBI, and potentially acute TBI, as well. Over 96% of the studies which compared SPECT to CT found SPECT identified lesions which were not evident on CT. For example, Abdel-Dayem et al., evaluated 228 subjects with mild-to-moderate TBI and found abnormally low perfusion in the frontal, temporal, and parietal lobes (60). A follow up study by Abu-Judeh et al. (61) in the same population found that abnormalities that were identified on SPECT were often not seen or were underestimated in magnitude on CT scan in those receiving both SPECT and CT (61). Ichise et al. (26) found similar discordance with 79% of SPECT abnormalities lacking a matching abnormality of CT and concordant lesions were larger on perfusion SPECT scan than on CT scan (26). Emanuelson et al. (73) showed that SPECT lesions were concordant in severe TBI, but SPECT was more sensitive than CT in mild TBI. In another study, SPECT scans in the acute setting detected abnormalities in 75% of patients who had amnesia symptoms, while the CT scans were read as normal (74). Given that CT scans have become the cornerstone of evaluating concussion and TBI in the acute setting wherein they readily reveal hemorrhage and fractures, it becomes important to recognize that CT scans fail to show functional deficits seen on SPECT for which there may be no structural correlates. Thus, CT scans for head trauma in the emergency department may be negative, but do not rule out future functional deficits. To this point, all subjects in the longitudinal study by Jacobs had negative CT scans in the acute setting (40, 64); however, a positive baseline SPECT scan had high sensitivity and specificity for persistent neurological symptoms (see below).

Similarly, SPECT is more sensitive for TBI than anatomical MRI across multiple studies. In a series of 13 patients with moderate TBI, Shin et al. (75) found that MRI was negative in 50% of the cases, while the SPECT scans analyzed with statistical parametric analysis were positive for brain injury in 100% of cases. Abu-Judeh et al. examined 228 patients with mild to moderate TBI in a retrospective review (61). Both CT and MRI within 2 weeks of injury were negative, while SPECT scans revealed frontal lobe injury in 24% of cases and temporal lobe injury in 13% of the cases. Likewise, Stamatakis et al. (47) examined 62 patients with TBI using MRI and SPECT, which were performed within 2 weeks of injury. Using statistical parametric analysis, they found SPECT detected more lesions and more lesion volume than anatomical MRI. Ichise et al. (26) found SPECT scans more sensitive than MRI as well, with 79% of SPECT abnormalities lacking a concordant MRI lesion. Conversely, MRI detected white matter hyperintensities which did not show a matching lesion on SPECT (26). Kinuya et al. (48) found SPECT detected hypoperfusion in 94% of cases wherein MRI scans were normal; however, cases of subdural hematoma did not show abnormal SPECT findings. SPECT findings correlated strongly with symptoms, such as personality change or amnesia.

#### Does SPECT Correlate With Neuropsychological Findings?

The current trend in neuropsychological assessment is toward the profiling of functional performance to detect TBI. The field is still hampered using many tests that are antiquated, excessively long, or of dubious psychometric quality (76). A neuropsychological assessment can consist of a multitude of tests; there are over 100 separate neuropsychological assessment tests that are frequently utilized in TBI cases (77). Because no single neuropsychological test is particularly sensitive for TBI (78, 79), they are generally used in batteries. However, a lack of consensus exists about which tests are appropriate to include in a battery (79). Accordingly, the choice of tests to include is subjective. In addition, variances between how individual neuropsychologists administer the tests, interpret the results, apply failure criteria and decide whether to test for effort are additional subjective variables (80). Moreover, comorbid conditions, such as pain, anxiety, depression, sleep disturbance, medications, and alcohol use can interfere with cognitive performance obscuring the effects associated with mild or even much more significant brain injury (77, 80). Lastly, the validity of a neuropsychological assessment battery is based on the norms, decision rules, false positives, false negatives, hit rates, and the compounding of these variables when multiple tests are combined in a battery (77). Hence, neuropsychological testing is not considered diagnostic for TBI (81).

Brain SPECT imaging provides neuropsychologists an objective way to address these problems. SPECT has, in fact, been correlated with several individual neuropsychological assessment tests such as the Wisconsin Card Sort (82–85), the Stroop Colored Word Test (86, 87), the Tower of London Test (88, 89), the Clock Drawing test (90, 91), the Test of Verbal Fluency (92) and the Auditory Verbal Learning test (93). SPECT perfusion patterns have also been found to correlate with the predicted localization of neurological damage, based on neuropsychological battery testing, in a number of conditions including Lyme's disease (94), Sjorgren's syndrome (95), Klein-Levin syndrome (96), obsessive compulsive disorder (97), migraine headaches (98), paraneoplastic encephalitis (99), cerebral microvascular disease (100), chronic alcoholism (101), Alzheimer's disease and dementia (102), and neurological impairment following coronary artery bypass grafting (103).

Additionally, 18 out of 21 cross-sectional studies (81%) included in a systematic review showed correlation between abnormal SPECT findings and neuropsychological deficits (44). This suggests that abnormalities found with brain SPECT can correlate with and therefore can be predictive of functional outcomes and/or neuropsychological test performance. Davalos and Bennett (54) examined this question based on three studies (26, 104, 105); however, two of the studies lacked a control group and one included only four patients. Nevertheless, they concluded that this correlation warranted further study and that the confound of depression, possibly secondary to the TBI, must be carefully considered. We do not disagree with these conclusions, given the extensive literature presented above.

#### Do SPECT Scans Predict Clinical Outcome?

Neuroimaging for head trauma serves multiple purposes. Establishing the presence/absence of TBI is first and foremost. Predicting clinical outcome is an important additional benefit which may or may not be realistic. For example, diffusion tensor imaging has not shown clear predictive utility for clinical outcome (106). Nevertheless, critics are quick to hold SPECT in rebuke for failing to absolutely predict clinical outcome. For example, a critical opinion piece on the use of SPECT to evaluate mild TBI by Wortzel et al. (55), which was poorly referenced, cites an unnamed study in which an abnormal scan was predictive of persistent clinical symptoms in 59% of cases. Presumably, this unnamed study is Jacobs et al. (40) based on the reference in Davalos and Bennett (54), which Wortzel et al. (55) were discussing when describing this unnamed study. However, this reference is flawed on several levels. First, Jacobs et al. (40) included subjects with both mild and moderate TBI. Second, this study was examining SPECT findings in the subacute setting (within 1 week) as predictors of persisting symptoms. Recovery was an expected outcome for a significant proportion of subjects. Thirdly, Wortzel et al. (55) ignore the further longitudinal data from Jacobs et al. (64). Therefore, these results will be detailed here.

Jacobs et al. (40) published the first part of a two-part longitudinal study of the correlation between acute SPECT scan findings and persistent neuropsychological symptoms in 1994. It is one of a number of studies which have documented the positive predictive value (PPV) and negative predictive value (NPV) of SPECT in the prediction of lasting neuropsychological effects of TBI. It was included in the TTASAAN report and is also likely the study referenced by Wortzel et al. (55) above. Jacobs et al. (40) conducted a scrupulous study of 67 subjects with acute TBI (42 moderate TBI, 25 mild TBI) who were then followed over the subsequent year (64). Furthermore, Jacobs et al. added an additional 69 subjects with mild TBI to the longitudinal sample. All subjects had baseline perfusion SPECT scans and CT scans obtained within 4 weeks (83% within 1 week) of the head injury event. All subjects had *baseline neuropsychological testing*.

Subjects with a positive finding on SPECT had a repeat SPECT scan at 3 months, 6 months, and at 1 year, while all subjects underwent repeat neuropsychological testing at 3 months, 6 months, and 1 year (64). These studies captured three key concepts in the evolution of mild TBI. First, a substantial proportion of patients with mild TBI recover over the course of the year, regardless of whether they have positive SPECT findings at baseline. The second, not every patient with mild TBI will have a positive SPECT scan. The third, the sensitivity and specificity of baseline SPECT for predicting persistent neuropsychological symptoms and findings can be calculated. In addition, the PPV and the NPV of a baseline SPECT scan in acute TBI was determined (Table 1).

**Table 1:** Outcome data from Jacobs et al. (40, 64).

**Number of months post-TBI**	**0**	**3**	**6**	**12**
Sensitivity	78%	91%	100%	100%
Specificity	61%	61%	53%	85%
Negative Predictive Value (NPV)	92% (3 mth) 100% (12 mth)	–	100%	100%
Positive Predictive Value (PPV)	44%	64%	52%	83%

*Patients who had a positive SPECT scan and clinical symptoms based on neurological examination, neuropsychological testing, and concussion questionnaires, had repeat SPECT scans at 3 months. If they had a positive SPECT scan at 3 months, then they had a repeat SPECT scan at 6 months, etc. The sensitivity and specificity of the baseline SPECT for clinical symptoms at 3 months was 78 and 61%, respectively. A persistently positive SPECT scan was predictive of persisting neuropsychological symptoms. A positive scan at 3 months had a sensitivity and specificity for persisting symptoms of 91 and 61%, respectively. If a scan was still positive at 12 months, the sensitivity and specificity of persisting symptoms was 100 and 85%, respectively. The PPV of the baseline SPECT scan was relatively low (44%) largely reflecting the degree of recovery seen in this population. Among patients with a negative SPECT scan at baseline, 92% were free of clinical symptoms by 3 months and 100% were free of symptoms at 12 months (NPV)*.

Based on this large sample of 136 subjects, a negative baseline SPECT scan was highly predictive of normal neuropsychological testing in the future. In other words, a negative SPECT scan shortly after initial injury predicts the absence of long-term functional deficits. This predictive value cannot be matched by other imaging modalities such as conventional CT or MRI. The positive predictive value of baseline SPECT scans was also quite high. An abnormal baseline SPECT scan that remained positive at 12 months predicted persistent neuropsychological deficits with a sensitivity of 100% and a specificity of 85% (64). This gives a strong argument for serial SPECT scans in cases with both positive baseline scan and neuropsychological symptoms. Similarly, a second prospective study by an independent group (107) found an abnormal baseline SPECT correlated strongly with abnormal neuropsychological testing in patients participating in a cognitive rehabilitation program.

Lastly, the lack of a randomized, placebo-controlled clinical trial of SPECT in the diagnosis of TBI is a deficit cited by *both critics* (54–56) *and proponents* (54, 108–110). The improbability of a gold-standard and randomizing patients to experience TBI make this critically needed study impossible. However, an additional, more nefarious, barrier has prevented such a study from occurring. One of us (TH) collaborated with Drs. Davalos and Bennett to implement a study to evaluate mild TBI with perfusion SPECT which addressed the concerns they raised in their review. Essentially, this study would have replicated Jacobs et al.' work (64) with better SPECT imaging, larger sample size, more extensive neuropsychological testing, and comparison to a carefully vetted normative dataset. Unfortunately, repeated funding attempts failed due to wholesale rejection of SPECT neuroimaging by grant reviewers. Comments such as, “The use of SPECT, a technique with poor spatial resolution, poses a concern.” and “use outdated techniques” peppered the peer review panel summary statements. Again, it seems odd that perfusion SPECT imaging is held to an unrealistic standard that fMRI, diffusion tensor imaging, FDG-PET, amyloid PET and other forms of neuroimaging do not meet. For example, numerous diffusion tensor imaging studies of mild TBI lack the rigorous criteria established by Davalos and Bennett (54) but these studies were still funded and published after these criteria were set (106, 111–118). For example, several diffusion tensor imaging studies lack control groups (115, 118), several have small sample sizes (111–115), several examined limited neuropsychological testing (112, 113, 115–119), and all lacked randomization.

Experts in the field are calling for greater collaboration between neurologists and nuclear medicine physicians to conduct the needed studies required to convince Neurology of the value of SPECT neuroimaging in the assessment of TBI (67, 108–110). Critics continue to claim that SPECT is not useful in the evaluation of TBI. Conversely, *according to the criteria set forth in the TTASAAN report* (1), the current literature supports the use of perfusion SPECT neuroimaging to evaluate TBI as a Type A Recommendation (strong positive recommendation) based on Class II evidence derived from multiple clinical studies with large N and control comparison groups (40, 47, 52, 53, 63, 64, 86) as presented above.

### Stroke

Stroke remains a leading cause of death and disability throughout the world. In 1996, the rate of new stroke cases was ~269 per 100,000 (120) and the prevalence was 988 per 100,000. In 2017, the rate of new stroke cases declined 11% to 150 per 100,000 (121), while the prevalence increased by 37% to 1363.5 per 100,000 (121). As a result, ~104.2 million people worldwide have experienced a stroke (122). Neuroimaging has a pivotal role in the assessment and clinical management of stroke.

The TTASAAN report (1) included 12 research studies on stroke, in which 10 were performed using single-head gamma cameras (25, 123–127). Since the publication of the TTASAAN report, significant advancements have occurred in imaging techniques to assess vascular anatomy and integrity. Magnetic resonance angiography (MRA), computed tomography angiography (CTA) and diffusion weight MR (DWI) have proven effective, safe, and rapid in modern hospital settings (128). However, much of the world does not enjoy the technical riches of hospitals in the United States. Thus, many of the MRI and CT techniques employed in stroke assessment are not available elsewhere. Perfusion SPECT remains a valuable tool for assessing acute strokes prior to administering intravenous tissue plasminogen activator (IV tPA) (129, 130), assessing subacute strokes for viability and size of the penumbra (131, 132), and assessing persistent stroke-related symptoms (133). Perfusion SPECT may also contribute to stroke risk assessment (134, 135).

Nevertheless, the first step in assessing a patient with symptoms of stroke or transient ischaemic attack (TIA) is a non-contrast CT scan. CT remains a rapid, safe, and effective means of determining if a stroke is hemorrhagic or not. The introduction of IV tPA as a treatment for dissolving and clearing clots, as well as clot retrieval techniques, has increased the need for *rapid* determination that a stroke is not hemorrhagic. Candidates for IV tPA need to be treated within 4.5 h of stroke onset. Thus, MR techniques, which are more rapid, have largely replaced perfusion SPECT scans in the assessment of acute stroke/TIA patients.

**Figure 6:**
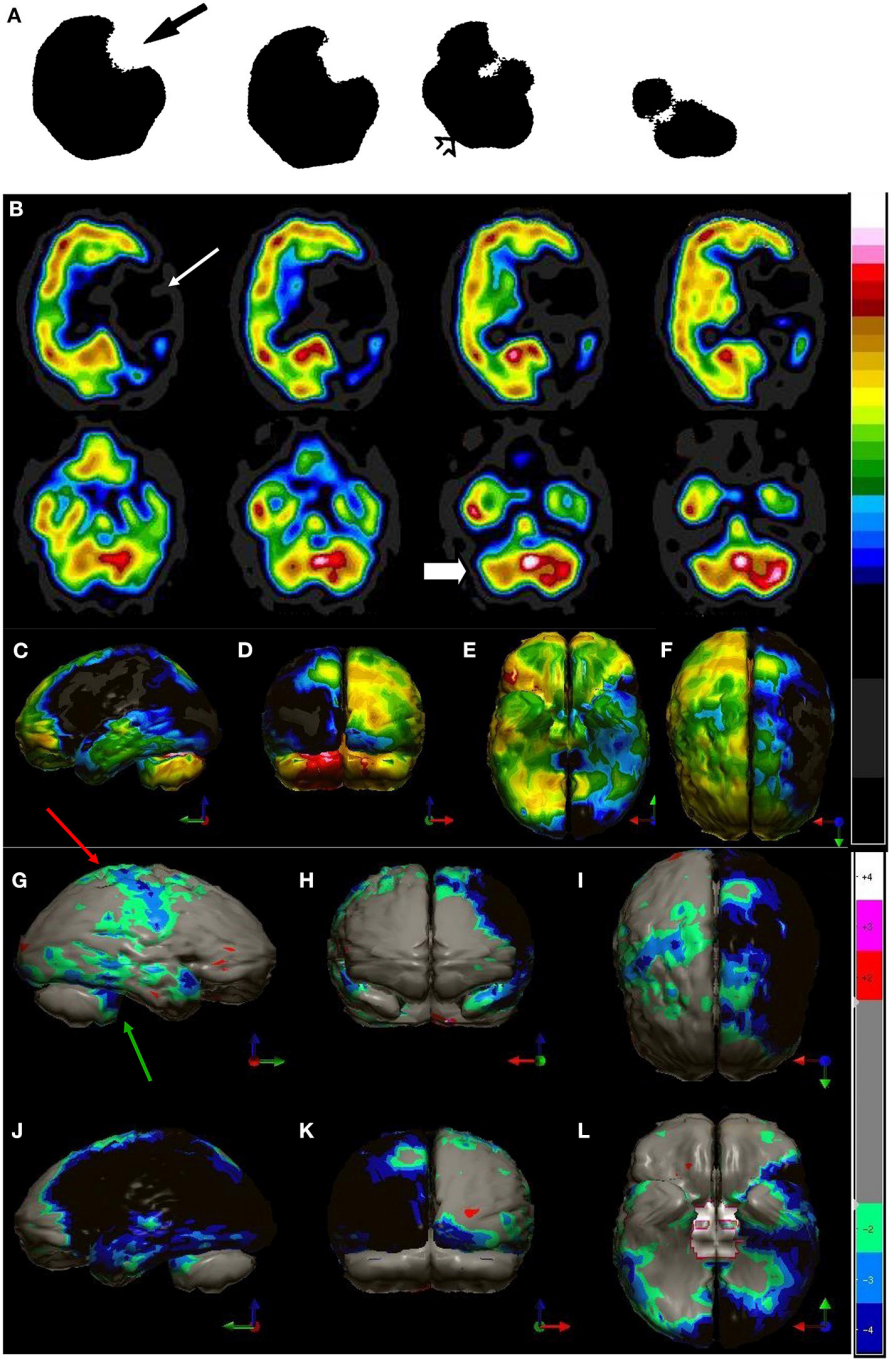
**(A)** Example of SPECT scan studies cited in TTASAAN report. Anatomical details are lacking. A ^99m^Tc-ECD perfusion SPECT scan from 1989 illustrating a left middle cerebral artery stroke (black arrow) with crossed cerebellar diaschisis (open arrow). This scan was performed on a single-head gamma camera [The figure was originally published in JNM. © SNMMI (25)]. **(B)** Modern 99mTc-HMPAO SPECT scan using a dual-head gamma camera illustrating a left middle and posterior cerebral artery stroke (white arrow) involving the left posterior frontal, temporal, parietal, and occipital cortices. Color scale is the same as in Figure 1B. Crossed cerebellar diaschisis is apparent (white block arrow), as well as involvement of the left thalamus and left basal ganglia. **(C–F)** left lateral, posterior, inferior (cerebellum removed) and superior 3-dimensional views, respectively. **(G–L)** Right lateral, frontal, superior, left lateral, posterior, and inferior (cerebellum removed) views of scan compared to normative database. Color scale is the same as in Figure 2D. Involvement of the contralateral cortex (red arrow) and the crossed cerebellar diaschisis (green arrow) are evident.

CT and diffusion/perfusion-weighted MRI have largely replaced perfusion SPECT in the assessment of subacute or chronic cerebrovascular disease. However, in certain conditions in which the variability of clinical presentation can be high (e.g., transient ischemic attacks, Moyamoya disease), SPECT may still offer value as part of the overall assessment plan. Perfusion SPECT neuroimaging is useful in delineating the extent of ischemic infarction and correlates well with severity of neurologic deficits and clinical outcomes. It may be useful in demonstrating the ischaemic penumbra at the margins of an ischaemic infarct, which may be salvaged with neuro-interventional procedures (132). This is due to the fact that perfusion SPECT imaging does not simply demonstrate the presence or absence of vascular occlusion. Rather, localized cerebral blood flow is regulated by the brain region itself. As the activity of a particular brain region increases, so does its need for oxygen and glucose. By a signaling mechanism involving neurons, glial cells, and the arterioles, the brain region calls for increase localized blood flow to meet its needs. Thus, perfusion SPECT neuroimaging shows active brain tissue, inactive brain tissue, and compromised brain tissue. Similarly, perfusion SPECT can be useful in assessing response to treatment or interventions (136, 137). A smaller volume of penumbra (138) and the absence of crossed cerebellar diaschisis (139) can be predictive of better clinical response. The technical improvements in gamma cameras and in post-processing software have markedly improved the resolution, anatomical detail, and information density of perfusion SPECT scans (Figure 6). 3-dimensional reconstruction allows more complete understanding of the stroke and penumbra volume. Statistical parametric analysis, particularly with comparison to a normative database, reveal details which might otherwise not be discernible (Figures 6G–L).

**Figure 7:**
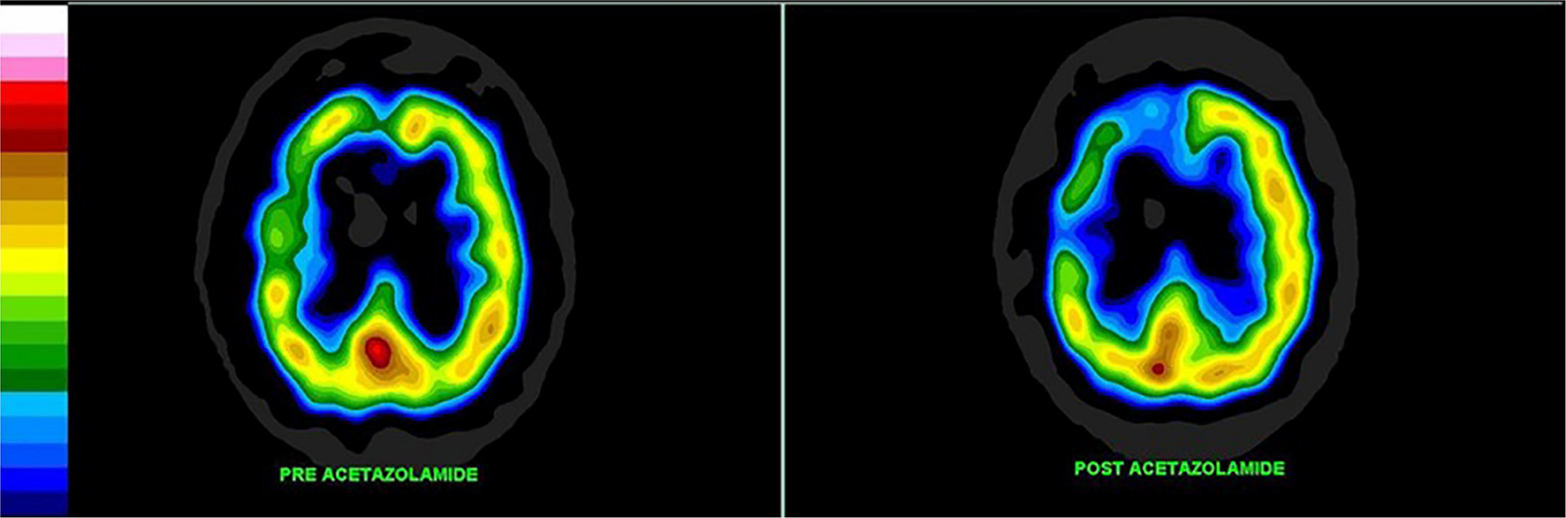
Patient with high grade carotid stenosis and multiple co-morbidities. The left hemisphere (on the right of the image) is able to augment its blood flow with acetazolamide, but there is restriction of flow to the right side leading to relative deterioration in right perfusion after acetazolamide administration, compared to the left. Color scale is the same as Figure 1B. Courtesy of J. Cardaci, MBBS, FAANMS, FRACP - Diagnostic Nuclear Imaging, Perth, West Australia; University of Notre Dame, Fremantle, Australia.

Cerebrovascular reserve capacity (CVRC) is an important parameter which guides treatment decisions in chronic cerebrovascular diseases. The cerebral circulation is complex with multiple arterial inputs to the Circle of Willis, with altered hemodynamics resulting from gradual occlusion of one of more vessels over time. In additional, there is highly responsive intracerebral autoregulation to maintain blood flow, particularly when cerebral perfusion pressure is reduced. Determining reserve capacity can be critical in assessing a patient for carotid endarterectomy or carotid stenting. Perfusion SPECT neuroimaging allows a global assessment of the integrated effects of hemodynamic factors, as it measures cerebral cortical and subcortical gray matter blood flow (140). Furthermore, dynamic assessment using intravenous acetazolamide or inhaled CO_2_ followed by perfusion SPECT provides an accurate measure of cerebrovascular reserve. Both acetazolamide and inhaled CO_2_ cause vasodilation of cerebral microvasculature (141). By challenging the vascular system with additional flow demands, these techniques reveal if the smaller arteries fed by the carotid arteries can support the flow demand (141). This is illustrated in Figure 7. This technique can be useful in decision making for patients with Moyamoya disease, as well.

### Epilepsy

The incidence of seizure disorders in the United States is 39 per 100,000, representing about 3.4 million cases (142). Approximately, one-third of all cases prove intractable or treatment-resistant—virtually unchanged from the time of the TTASAAN report, despite 25 years of new anticonvulsant medications. Surgical or laser ablation of the seizure focus/foci remains the most effective treatment in these cases. Ictal and inter-ictal perfusion SPECT scans remain an integral part of the pre-surgical evaluation of such cases (143–145).

The TTASAAN report (1) included 17 papers and reviews on the use of perfusion SPECT scans in the evaluation of epilepsy with a total of 182 patients (146–151). Based on this very preliminary data, the conclusion was that perfusion SPECT performed during the seizure event (ictal) could localize the seizure focus in 71–93% of cases with a positive predictive value of 95% (1).

In the last decade alone (2012–2021), there have been over 181 research articles and reviews containing a total of 8,516 patients on the topic of the utility of perfusion SPECT scans in the evaluation of epilepsy. The predominate topic was pre-surgical planning; however, diagnosis of pseudoseizures and seizures due to other medical conditions were also topics. In addition, over 85 cases studies of 1–3 patients each covered the utility of perfusion SPECT imaging in the *differential diagnosis* of a wide variety of medical causes of seizures, including TBI (152), tuberous sclerosis (153), vascular disease (154), neoplasms (155), systemic lupus (156), rare autoimmune disorders [e.g., anti-NMDA receptor autoimmune encephalitis (157, 158), hyperglycemia (159), and Lewy Body dementia (160)].

**Figure 8:**
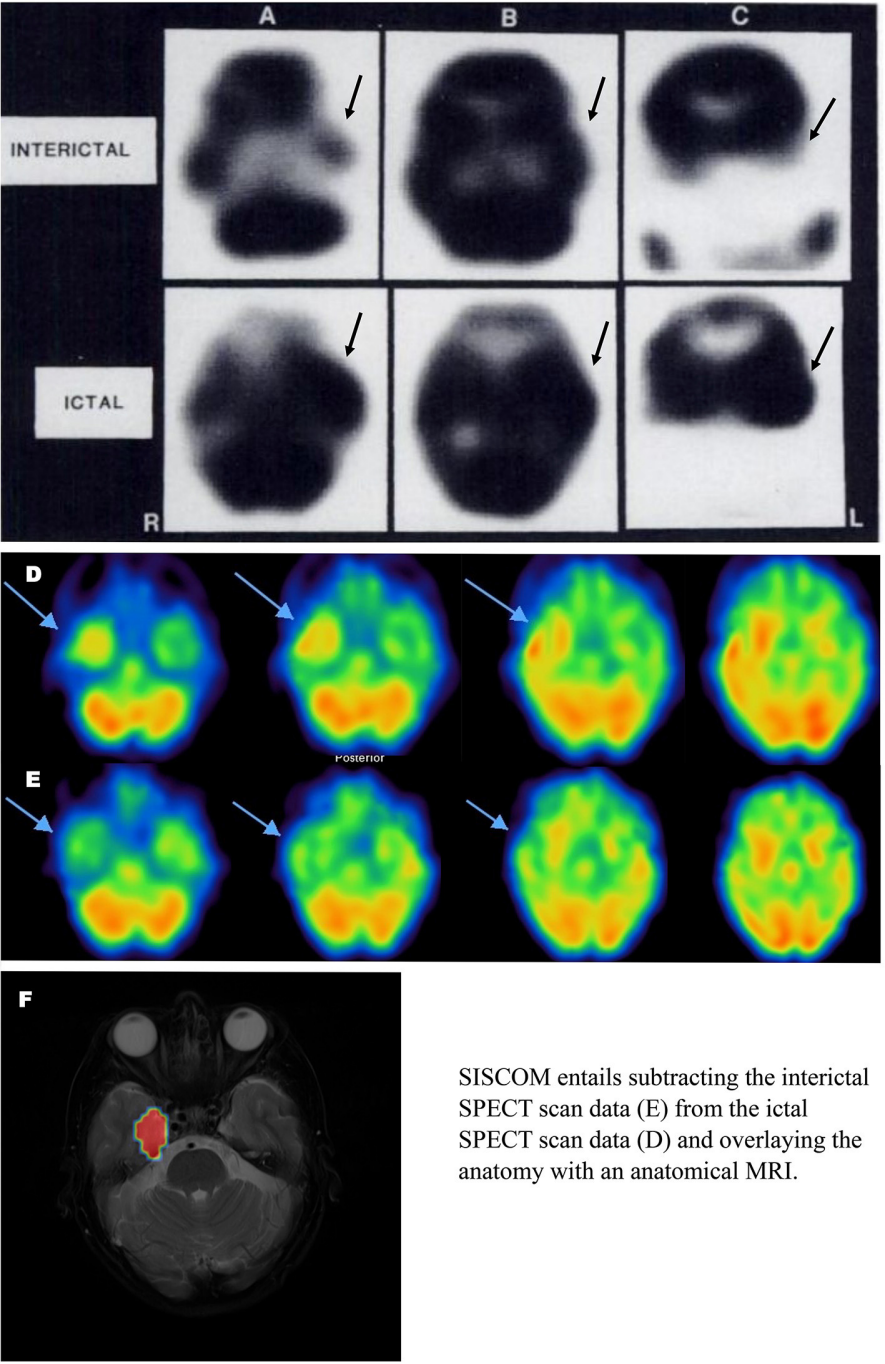
Ictal and Interictal SPECT **(A–C)** Example of SPECT scan studies cited in TTASAAN report. Perfusion tracer is iodinated N,N,N'-trimethyl-N'-(2-hydroxy-3– methyl-5-iodobenzyl)-1,3-propane-diamine (HIPDM). Anatomical details are lacking [The figure was originally published in JNM. © SNMMI (149)]. **(D,E)** Modern 2021 HMPAO SPECT perfusion scans for seizure localization. **(D)** Ictal scan. **(E)** Interictal scan. **(F)** SISCOM image wherein interictal scan data is subtracted from ictal scan data and the resulting data is overlayed on an anatomical MRI. Images courtesy of– Leonard Numerow MD FRCP(C), Radiology and Nuclear Medicine, Cumming School of Medicine, University of Calgary, Calgary, Alberta, Canada.

Two significant advancements in perfusion SPECT imaging, beyond the stabilization of HMPAO and the improvements in both gamma camera technology and post-processing software, have impacted the utility of SPECT imaging in the localization of seizure foci. The first is subtraction ictal SPECT co-registered to MRI (SISCOM). In essence, the interictal data is subtracted from the ictal data to reveal a focus or foci of increased perfusion during the ictus. The focus/foci are then mapped onto the patient's anatomical MRI (161–164). A retrospective study of 90 patients undergoing surgical resection of seizure foci between 1995 and 2013 revealed SISCOM predicted patients being seizure-free up to 5 years after ablative surgery (69.2%), comparable to FDG-PET localization (162). Smaller studies have found similar positive predictive value [76.9% - (165)]. The ability of SISCOM to accurately localize a seizure focus ranged from 73 to 85% (mean 77.3%) across multiple studies (164–172). A meta-analysis of 11 studies found SISCOM accurately localized seizure focus in 85.9% of cases and was concordant with EEG data in 65.3% of cases (166). SISCOM had an odds ratio of the patient being seizure-free post-operatively of 1.90 in non-concordant cases and of 6.23 in concordant cases (166). Recently, the diagnostic accuracy and predictive value of ictal perfusion SPECT was compared to magnetoencephalography (MEG) in a group of 158 surgical candidates. The accuracy of ictal SPECT was 78.57% in localizing the seizure focus, while the accuracy of MEG was 74.26% (1, 171). The odds ratio for a patient being seizure-free after surgery was 5.0 and 2.43 for ictal SPECT and MEG, respectively (171). Figure 8 illustrates the advances in perfusion SPECT imaging, including the SISCOM technique for localizing seizure foci.

The second advancement has been the use of automated injectors to reduce the lag time between the onset of seizure activity and the injection of the tracer (173, 174). Automated injectors can reduce lag time from 60 s with hand administration to 18.5 s using an automated method (175). Since seizure activity is initially limited to the focus/foci, but then spreads to adjacent or even contralateral brain areas, capturing the perfusion pattern early increases the accuracy of the localization by perfusion SPECT (176, 177). For example, using automated injectors increased localization of seizure foci from 62.9 to 81.8% (175).

Perfusion SPECT also has proven useful in cases of non-convulsive status epilepticus (NSE) among medically complicated patients (178). NSE can prove to be a challenging diagnosis and is more likely in critically ill patients with multiple overlapping diagnoses. In a group of 55 patients, initial EEG had a sensitivity of 61.1% and a specificity of 89%. In contrast, a perfusion SPECT had a sensitivity of 80.5% and a specificity of 89.5% in diagnosing NSE (178).

### Dementia and Alzheimer's Disease

The TTASAAN report (1) included 25 research articles and reviews on dementia (179–185). All of the studies were conducted using single-head gamma cameras, except one which used an annular crystal. Since the TTASAAN publication, over 600 research studies have been published on the subject of perfusion SPECT in the evaluation of dementia.

Currently, the most reliable neuroimaging findings for Alzheimer's disease (AD) are the observed decreased metabolic activity and associated decreased perfusion of the posterior parietal and temporal lobes bilaterally and the posterior cingulate gyri bilaterally with relative sparing of the basal ganglia, thalamus, and primary sensory-motor cortex (186–191). The early SPECT perfusion studies of AD relied on single-headed or low-resolution gamma cameras (192–194). Nevertheless, a meta-analysis of these early studies concluded perfusion SPECT has a sensitivity of 74% and a specificity of 81% for the differentiation of AD from elderly controls (195).

Since 2009, there have been three meta-analyses (196–198), a comprehensive review with meta-analysis (191), and a systematic review (199) addressing the clinical utility of perfusion SPECT neuroimaging in AD. Yuan et al. (196) found SPECT had a sensitivity of 84% and a specificity of 70%, while Bloudek et al. (197), performing a meta-analysis on selected research from 1990 to 2010, concluded that perfusion SPECT had a sensitivity of 79% and a specificity of 84%. Subsequently, Frisoni et al. examined 32 studies and found SPECT had a sensitivity of 76% and a specificity of 84% (198). In contrast, the sensitivity of FDG-PET ranged from 76 to 89% and the specificity ranged from 74 to 85% (191). The meta-analyses described above all co-mingled SPECT studies using single-headed gamma cameras with studies using multi-headed gamma cameras. Moreover, the summary sensitivity/specificity figures result from combined comparisons of AD cases vs. mild cognitive impairment (MCI), fronto-temporal dementia (FTD), vascular dementia (VaD), dementia with Lewy Bodies (DLB), and healthy elderly controls. As a result, these meta-analyses are not consistent with the conclusions of others (200, 201). For example, Bonte et al. (201) found that based on correlation to autopsy data, decreased posterior cingulate perfusion alone has a positive predictive value of 93% and a negative predictive value of 81%.

**Figure 9:**
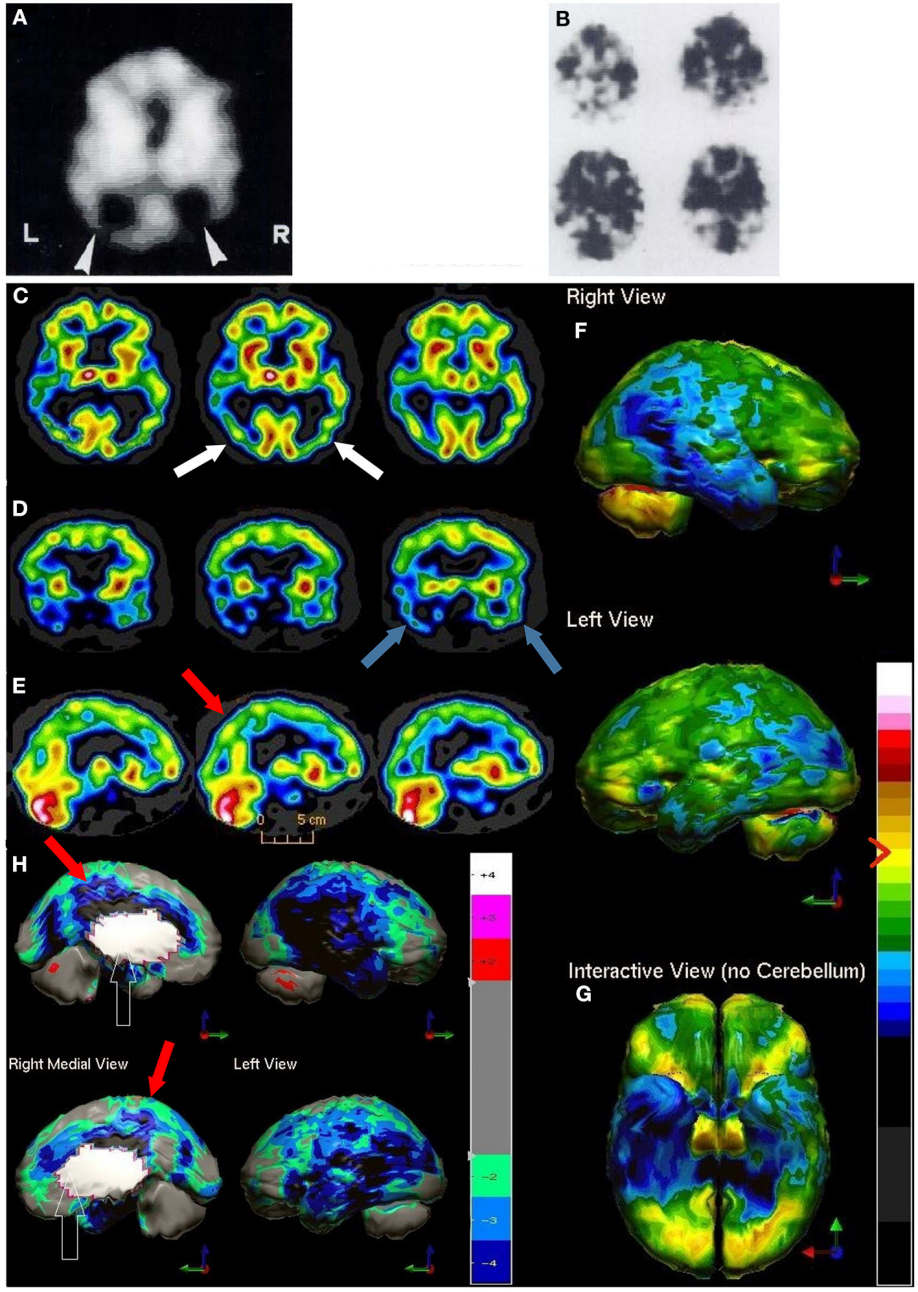
**(A,B)** Examples of SPECT scan studies cited in TTASAAN report. Anatomical details are lacking. **(A)** An ^99m^Tc-HMPAO perfusion SPECT performed in 1988 using a single head gamma camera illustrated a 75-year-old male with severe dementia and showed decreased perfusion of the bilateral parietal cortices [The figure was originally published in JNM. © SNMMI (202)]. **(B)** Example of ^133^Xenon perfusion SPECT scan obtained with a ring-type tomogram circa 1988 [The figure was originally published in JNM. © SNMMI (180)]. C-H) Modern ^99m^Tc-HMPAO SPECT scan performed on a dual-head camera illustrating Alzheimer's disease. **(C–E)** Horizontal, coronal, and sagittal tomograms, respectively, show decreased perfusion of the bilateral parietal cortices (white arrows), bilateral temporal cortices (blue arrows) and the posterior cingulate gyri (red arrow). Color scale is the same as in Figure 1B. **(F,G)** 3-D representation of SPECT scan data showing right lateral, left lateral **(F)**, and inferior views **(G)**. In the 3-D representations the asymmetry is much better seen with greater involvement of the right parietal and temporal lobes. **(H)** The patient's data is compared to a normative database using Segami Inc. Oasis software. The color scale is the same as in Figure 2D. Areas of relatively decreased perfusion are much more evident. The hypoperfusion of the posterior cingulate gyri (red arrows) is much better demonstrated.

A more recent meta-analysis was conducted which grouped data based on camera type and comparator group (FTD, VaD, DLB, MCI, healthy elderly control) (191). When differentiating AD from healthy elderly controls, studies using relatively low-resolution, single-headed gamma cameras yielded an overall sensitivity of 84% and an overall specificity of 83% (187, 200, 202–211). Studies utilizing multi-headed gamma cameras and often quantitative analysis (91, 186, 212–231) yielded a modest, but clinically significant, increase in overall sensitivity to 89% and overall specificity to 89%. Figure 9 illustrates the advancements in the images produced by perfusion SPECT imaging including statistical parametric analysis to an age-matched normative database as depicted for AD.

Longitudinal clinical studies have corroborated that perfusion SPECT scans can predict the advancement of SPECT-identified AD to autopsy-proven AD, as well as the progression of MCI to AD. Jobst et al. (210) followed 200 patients with dementia and 119 controls over 7 years. Seventy patients were autopsied, and baseline clinical evaluation alone yielded a sensitivity of 93% and a specificity of 46% in predicting histopathology consistent with AD, while baseline SPECT scans combined with clinical diagnosis did not change the sensitivity, but increased the specificity to 84%. Hanyu et al. (200) examined a group of 219 patients and were able to distinguish 56 cases that would progress to AD based on decreased perfusion of the temporal and parietal lobes (sensitivity 82%, specificity 89%). Matsuda et al. (232) utilized Z-score analysis to demonstrate that decreased posterior cingulate/precuneus perfusion could distinguish 40 patients with MCI suspected of being the AD-type from 40 controls with an accuracy of 86%. Taken together, studies of perfusion SPECT in the diagnosis of AD with comparison to a longitudinal clinical course and/or histopathology demonstrate sensitivity in the range of 82–96% and specificity in the range of 83–89% (191).

SPECT neuroimaging can be extremely helpful in the evaluation of dementia of a vascular origin (VaD). VaD can show widely varying regional blood flow patterns, reflecting its variable vascular source (233). As such, there is not a single characteristic pattern of perfusion or metabolic activity that identifies VaD dementia (191, 233, 234). However, certain features are highly suggestive of vascular dementia (235), such as hypoperfusion of the anterior cingulate gyrus (which mitigates relatively against AD) or reduced perfusion of the pulvinar of the thalamus as seen in subcortical VaD (191). Rather, it is often the varied, bilaterally disparate, and irregular pattern that aids in the diagnosis of vascular dementia. A meta-analysis of perfusion SPECT studies utilizing single-headed gamma cameras with only visual interpretation to distinguish AD from VaD found sensitivity to be 70% and specificity to be 76.6% (191, 236–240). In contrast, the use of multi-headed gamma cameras and quantitative analysis improved sensitivity to 90%, while specificity remained slightly better than 76% (191, 218, 230, 241, 242). Neuroimaging data suggest that different types of VaD can be distinguished by SPECT; indeed, some authors feel that SPECT is more helpful in the diagnosis of different forms of vascular disease than PET (191, 233).

Frontal temporal dementia (FTD) can be characterized on functional brain imaging by decreased function and associated hypo- perfusion in the frontal lobes, caudate nuclei, and anterior temporal lobes (243–247). Hypoperfusion also can be found in the anterior cingulate gyrus. In FTD, perfusion is generally spared in the posterior cingulate gyrus/precuneus (91, 201, 248, 249). For example, Bonte et al. (186) followed 54 patients to autopsy, and a SPECT scan up to 1 year prior to death predicted the histopathology with a 96% sensitivity and an 84% specificity. The meta-analysis of all studies utilizing single-headed gamma cameras yielded a sensitivity of 71.5% and a specificity of 78.2% in the differentiation of AD from FTD (191, 244, 250–252). In contrast, a meta-analysis of studies utilizing multi-headed gamma cameras and quantitative analysis found the sensitivity was 96% and the specificity was 80% in differentiating AD from FTD (91, 191, 246, 249, 253). For example, in a careful study with quantitative analysis of SPECT data from a multi-headed gamma camera compared to autopsy findings, the temporal-parietal and posterior cingulate gyrus hypoperfusion had high sensitivity and specificity for distinguishing AD from FTD (91).

Similarly, the differentiation of AD from Lewy Body dementia (DLB) by perfusion SPECT imaging has benefited from improved hardware and analysis techniques. Shimizu et al. (254) examined the differentiation of DLB and AD using statistical parametric analysis to evaluate cortical, as well as deep nuclei, perfusion. They found that while hypoperfusion in the occipital lobe was predictive of DLB, the additional findings of increased perfusion in the thalamus and bilateral striatum strengthened the accuracy of the diagnosis of DLB. Sato et al. similarly found increased perfusion of the thalamus and striatum differentiated DLB from AD (255). Goto et al. (256) also found striatal parameters useful in differentiating early mild DLB from early mild AD. They found striatal volume to be reduced in early DLB, along with reduced occipital perfusion. The sensitivity of these parameters was 89%, while the specificity was 84%.

The accurate and early identification of Mild Cognitive Impairment (MCI) with perfusion SPECT has been examined extensively in several longitudinal studies. A total of 495 patients with MCI have been followed over 2–3 years [one study to 5 years (257)] in 10 longitudinal studies that included a baseline SPECT scan. All the studies used multi-headed gamma cameras and quantitative analysis (191, 257–266). Overall, 43% of MCI patients showing decreased perfusion in the: 1) posterior cingulate gyri, 2) posterior parietal cortices, and/or 3) temporal cortices converted to AD with an average conversion rate of 20% per annum. The remaining patients showed no change in cognition (stable MCI). Thus, the key SPECT findings found in AD were already present 2–3 years before the onset of the clinical symptoms of AD (191, 228, 229, 266). With quantitative analysis, the predictive value of perfusion SPECT in MCI can be increased considerably. Quantitative analysis yielded a sensitivity of 97%, a specificity of 100%, and an accuracy of 99% in one study (228). SPECT outperforms clinical assessment for MCI, which is generally 49–63% sensitive and 89–94% specific (267). Using older, single-headed gamma cameras and visual inspection, several studies found that SPECT can differentiate MCI with a sensitivity of ~84% and a specificity of 83% (191). In contrast, multi-headed gamma cameras and quantitative analysis yields sensitivity and specificity exceeding 97% depending on the study (191, 228, 268).

Given that gamma cameras are much more readily available than PET scanners in much of the world and perfusion SPECT scans can be performed at a much lower cost than FDG-PET scans (191, 269–271), it is important to assess the value-added benefit of choosing PET or SPECT imaging (See Table 2). A relatively small number of studies have examined the distinction between SPECT and PET imaging in the evaluation of AD in the same patients. For example, a pivotal study by Herholz et al. (269) compared FDG-PET and perfusion SPECT in the same 26 patients with probable AD. In the key areas of the temporal, parietal, and posterior cingulate cortices, FDG-PET and SPECT yielded corresponding findings (*r* = 0.90) using statistical parametric analysis. Silverman (188) summarized the comparison of PET and SPECT well, noting that the sensitivity and specificity of PET and SPECT have considerable overlap. Similarly, a systematic review of studies comparing perfusion SPECT and FDG_PET in the diagnosis of AD and other dementias (199) found the two techniques quite comparable in terms of accuracy of diagnosis. The application of quantitative analysis greatly enhances both PET and SPECT. With appropriate quantitative analysis, both neuroimaging modalities can achieve high sensitivity and specificity in the diagnostic evaluation of AD and MCI, and in the differentiation of AD from LBD. Specifically, FDG-PET was found to predict the conversion from MCI to AD with a sensitivity of 70–90% and a specificity of 82.4–90% depending on the study (191, 196, 272, 273), while perfusion SPECT scans predict the conversion from MCI to AD with a sensitivity ranging from 89 to 97% and a specificity of 89–100% depending on the study (191, 196, 221, 225, 263, 274). Amyloid PET scans also should be included in this discussion given the growing number of amyloid tracers available. PET scans using amyloid-specific markers, such as florbetaben or florbetapir, yield very high sensitivity. A widely held belief is that a positive amyloid scan is 100% predictive of a progression to AD. However, specificity varies with age. A caveat for amyloid markers, regardless of the marker, is non-specific binding does occur and increases with age. Such non-specific binding occurs in ~20% of 60-year-old controls, but this increases with age to ~40% of 80-year-olds (275, 276). A recent Cochrane Review (277) found that amyloid markers predicted the progression from MCI to AD over a 4-year period with a sensitivity of 67% and a specificity of 71%. Shorter follow-up time courses yielded different metrics with a sensitivity of 89% and a specificity of 58% at 2 years (277).

**Table 2:** Sensitivity and specificity of perfusion SPECT neuroimaging for differentiating Alzheimer's disease (AD) from controls, vascular dementia (VaD), and fronto-temporal dementia (FTD).

	**SINGLE-HEAD**		**MULTI-HEAD**	
	**Sensitivity**	**Specificity**	**Sensitivity**	**Specificity**
AD vs. Control	84%	83%	89%	88%
AD vs. VaD	70%	76.6%	90%	76.4%
AD vs. FTD	71.5%	78.2%	96%	80%
MCI → AD	84%	83%	89–97%	89–100%
FDG MCI → AD	–	–	89%	84%
Amyloid PET MCI → AD	–	–	67–87%	51–71%

*Also shown are the sensitivity and specificity for predicting the progression of mild cognitive impairment (MCI) to AD. Results for perfusion SPECT imaging are separated based on data derived from studies utilizing single-headed vs. dual-headed cameras. Also shown are sensitivity and specificity of predicting the progression from MCI to AD for fluoro-deoxyglucose positron emission tomography (FDG-PET) and for amyloid tracer PET*.

### Neuropsychiatric Conditions

#### Neurotoxicity

While not addressed in the TTASAAN report (1), perfusion SPECT neuroimaging has proven valuable in the evaluation of encephalopathy and of psychiatric cases wherein toxic encephalopathy may be a contributing factor to symptomatology. Solvent-induced encephalopathy has been investigated with perfusion SPECT (109, 278–280). Perfusion SPECT revealed diffuse hypoperfusion in 94% of cases in one study (279), while CT and MRI identified abnormalities in only 7 and 29%, respectively. Perfusion SPECT reveals diffuse hypoperfusion in metal toxicity (281), mold toxicity (282), and other toxin exposure (109, 283, 284), including recreational toxins (284–290). SPECT is also beneficial in the identification and grading of severity of hepatic encephalopathy due to ammonia toxicity (291–295), even in mild cases (296), as well as for tracking progress (295). Carbon monoxide poisoning is characterized by decreased perfusion of the bilateral frontal cortex, bilateral temporal cortex, and the globus pallidus (297–301) and perfusion SPECT has been used to document these findings. To the extent that toxicity can induce symptoms which can be confused with psychiatric symptoms, perfusion SPECT can lead to clarification of the diagnosis and detoxification, rather than psychiatric pharmacology. For example, in a patient with signs of decreased frontal lobe function (e.g., ADHD, aggression, anti-social personality disorder) a perfusion SPECT finding of diffuse hypoperfusion increases the likelihood of toxicity as the true cause of the frontal lobe dysfunction (109, 110, 302).

Specifically concerning recreational drugs, perfusion SPECT imaging reveals diffuse hypoperfusion throughout the cerebral cortices, but predominately in the frontal and temporal cortices (303–307). Dopamine transporter SPECT scans (DaTscans) demonstrate the presence and availability of dopamine transporter sites (DAT). A recent meta-analysis demonstrated reduced DAT density in the striatum, as well as all areas of cortex, among abusers of cocaine, methamphetamine, and amphetamine, consistent with down-regulation of the dopamine system among stimulant abusers (308, 309). In contrast, nicotine abusers did not show decreased DAT availability (309). Similar decreases in DAT density have been reported in opiate abusers (310). Similar findings have been reported with PET tracers for the DAT (311).

Carbon monoxide (CO) poisoning warrants a separate description because of its distinct findings on perfusion neuroimaging. In addition to the acute toxic effects of CO, victims can experience delayed neurological symptoms. Often significant recovery from the acute neurological insult can occur followed by a dramatic decline in neurological function, including loss of cognitive function, behavioral changes, gait disturbances, Parkinsonian symptoms, incontinence, and aphasia (301, 312–315). In acute CO toxicity, perfusion SPECT neuroimaging can reveal decreased perfusion in the basal ganglia (300, 316–318), along with decreased perfusion of the bilateral frontal, temporal, and (to a lesser extent) parietal cortices (300, 301, 316, 318). The degree of cortical hypoperfusion can be correlated with the degree of neuropsychological impairment (300, 317, 319, 320). Often CT scans are normal, with 0% sensitivity vs. 70% sensitivity for SPECT scan in one study (309). Perfusion SPECT scans were also more sensitive (66%) than EEG (25%) in the acute setting (321).

Similarly, perfusion SPECT neuroimaging has proven useful in the evaluation of delayed neuropsychiatric sequalae of CO poisoning, as well in assessing response to treatments (301, 314, 315, 322–325). Hypoperfusion of the basal ganglia have been correlated with the well-established finding of T2 hyperintensities in the globus pallidus by MRI (314, 320, 324). Similarly, hypoperfusion in bilateral frontal and temporal cortices has been correlated with reduced anisotropy by diffusion tensor imaging (325). The degree of cerebral hypoperfusion has been correlated with cognitive impairment (299, 301, 320) and with symptomatic improvement following treatments, such as hyperbaric oxygen therapy (314, 315, 322, 325). While bilateral globus pallidus injury, edema, and necrosis are considered a hallmark MRI finding in chronic CO toxicity, not every patient manifests these severe findings. For example, in a series of 21 patients with chronic CO toxicity, 38% had abnormal MRI findings, while 67% had abnormal perfusion SPECT scans (319). This question was again examined with a group of 30 patients with chronic CO toxicity (320). MRI demonstrated greater sensitivity, while perfusion SPECT had higher specificity, positive predictive value, and negative predictive value for persistent neuropsychiatric symptoms (320).

#### Psychiatric Indications

The use of perfusion SPECT neuroimaging for psychiatric indications has increased significantly over the past two decades. Unfortunately, it has not been widely adopted in either nuclear medicine or psychiatry for several reasons. First unlike neurological diagnoses, which are ultimately verifiable by biopsy, there are no recognized histopathological markers for psychiatric diagnoses, which is the case with Alzheimer's disease, stroke, or other dementias. Rather, psychiatric disorders are defined by the Diagnostic and Statistical Manual of Mental Disorders Version 5 (DSM-5). Psychiatric diagnoses are based not on pathology but upon a constellation of symptoms. However, there is often tremendous range in how patient's symptoms present. For example, using the DSM-5 (326) diagnostic criteria for Major Depressive Disorder (110, 327), there are over 20 possible distinct phenotypes of this single diagnosis. The failure of clinical trials to effectively treat depression has led experts to state

*“…that major depressive disorder is biologically heterogeneous, such that different treatments differ in the likelihood of achieving remission in different patients” (328)*.

Since psychiatry has difficulty establishing correct diagnoses and therapies, it is not surprising that perfusion SPECT has not established pathognomonic perfusion patterns.

Second, comorbidity is the rule rather than the exception in psychiatric conditions. It is difficult to identify a patient with pure bipolar disorder, for example, when the comorbidity of attention-deficit-hyperactivity-disorder (ADHD) occurs in ~57% of adult bipolar patients (329) and up to 98% of pediatric bipolar cases (330). Similarly, in depression, in addition to having a multiplicity of presentations, many depressed patients also are comorbid for anxiety in up to 60% of cases (327, 331, 332). Patients with ADHD frequently have coexisting mood disorders (59%), anxiety, oppositional disorders, or learning disorders (327, 333–336). For all these reasons, it is highly unlikely that a pathognomonic finding or a “neuroimaging fingerprint” will be found for depression, anxiety, bipolar disorder, obsessive compulsive disorder (OCD), or ADHD. Indeed, these complexities result in widespread failure of the diagnostic criteria in field testing (337).

Despite these limitations, a substantial body of research literature exists for brain perfusion SPECT in the evaluation of psychiatric disorders. In addition, hundreds of thousands of perfusion SPECT scans have been performed worldwide since the development of the technology in the 1990's. Certain patterns or highly consistent findings have been replicated in the research literature. Findings with extensive clinical correlation will be noted here.

### Attention Deficit Hyperactivity Disorder

Decreased frontal lobe perfusion is a consistent finding in ADHD across multiple SPECT studies (327, 338–345) and confirmed by multiple functional MRI studies (346, 347) and infrared spectroscopy (348). For example, SPECT scans of medication-naïve children with ADHD (*N* = 40) were compared to normal controls using statistical parametric analysis (338). Decreased perfusion was found in the prefrontal cortex, orbitofrontal cortex, and middle temporal gyri, while increased perfusion was found in the somatosensory cortex and anterior cingulate gyri (338). With stimulant treatment, perfusion increased in the prefrontal cortex (338, 343) corresponding with clinical improvement of ADHD symptoms. Clinical experience has heavily supported these findings (109, 302, 349).

Perfusion SPECT neuroimaging also is beneficial in the differential diagnosis of ADHD. Since inattention, impulsivity, and hyperactivity are non-specific signs of frontal lobe dysfunction, it is not surprising that toxicity, concussive brain injury, incipient bipolar disorder, infection, and inflammation can produce similar symptom constellations as ADHD. SPECT can reveal these alternative causes (109, 110, 302, 349). For example, infection or toxicity likely will appear as diffuse hypoperfusion as elaborated in the section on Neurotoxicity above. Brain injury will likely appear as an asymmetrical area of hypoperfusion. In contrast, bipolar disorder will present as described in the next section.

#### Bipolar Disorder

In contrast, bipolar mania, which can present symptomatically like ADHD, often demonstrates increased perfusion in the frontal cortex, particularly the dorsolateral prefrontal cortex and possibly greater on the left (350, 351). Patients with bipolar mania also typically do not show the decrease in prefrontal perfusion unless they have comorbid ADHD as described above (109). While the total number of subjects studied in ADHD and bipolar disorder number <200, the clinical experience across hundreds of thousands of scans supports the correlation of these disease processes with these perfusion patterns.

Increased and asymmetric perfusion of the thalamus may serve as a possible endophenotypic pattern of bipolar disorder in the manic or euthymic states (352–354). Bipolar depression may be similar to unipolar depression in terms of decreased frontal cortex perfusion (355), but it is possible the two can be distinguished by differences in the perfusion of the thalamus and basal ganglia in the depressed state. Perfusion, whether measured by SPECT or fMRI, is increased in the thalamus in bipolar disorder (350, 353–357). It must be emphasized that these types of endophenotypic patterns may not be evident upon visual inspection of tomographic data for an individual SPECT scan. Rather, these findings may only be manifest in the statistical comparison of perfusion data to normative databases.

### Depression

Over 150 studies of perfusion SPECT imaging in depression containing more than 12,100 subjects have been completed. A consistent finding in early SPECT (Xenon or HMPAO) studies of depression was decreased perfusion in the frontal, and often temporal, cortices, as well as the superior anterior cingulate gyri (358–361). Later, two distinct patterns of perfusion were recognized – decreased perfusion in typical and melancholic depression and increased frontal lobe perfusion in atypical depression (362–365). Increased perfusion in the subgenual anterior cingulate gyrus in treatment-resistant depression was first described by Goodwin et al. (366) and has been recognized as a hallmark sign of treatment resistant depression, subsequently (365, 367, 368). Remission or response to treatment is characteristically followed by increased perfusion in the affected areas (366, 369, 370). Response could be predicted by the degree of frontal hypoperfusion and of subgenual hyperperfusion. Notably, response to serotonin reuptake inhibitors was predicted by higher frontal and cingulate perfusion (368, 371, 372), while response to electroconvulsive therapy (ECT) or transcranial magnetic stimulation (TMS) was predicted by lower frontal and cingulate perfusion. Increased metabolic activity and perfusion in the thalamus (373, 374) is also a frequently reported finding in unipolar depression. Increased symmetrical perfusion of the thalamus has been consistently seen by clinicians on tens of thousands of perfusion SPECT scans.

### Obsessive Compulsive Disorder

OCD is considered to result from an abnormal overactivity of a circuit involving the frontal cortices, anterior cingulate gyri, caudate nuclei and the thalami (375). Increased perfusion of the caudate nuclei and the anterior cingulate gyri have been reliable perfusion SPECT findings across 10 studies involving 196 subjects with OCD vs. 117 controls (376–385). Similar increased metabolism in these same areas has been found in studies utilizing FDG-PET (386, 387) and fMRI (388, 389). These findings were recently reviewed by Hazari et al. (390). Moreover, these findings have been consistently seen by clinicians on tens of thousands of perfusion SPECT scans.

### Post-traumatic Stress Disorder

The symptom overlap between post-traumatic stress disorder (PTSD) and traumatic brain injury has complicated the correct diagnosis, particularly among military personnel (391, 392). Perfusion SPECT studies and FDG-PET studies have made similar findings in PTSD. Increased perfusion of the caudate nuclei is often found in PTSD (52, 53, 393). Another SPECT study showed that compared to controls, PTSD patients had increased cerebral blood flow in the limbic regions along with decreased perfusion in the superior frontal, parietal, and temporal regions (394). Systematic analyses of multiple regions of the default mode network involving 10,076 patients with TBI, PTSD, or both compared to 11,263 controls, revealed that PTSD resulted in increased perfusion in the basal ganglia, cingulate gyri, thalami, prefrontal cortices, and medial temporal cortices in both military (52) and civilian (53) populations. Provocation studies using perfusion SPECT, perfusion PET, and fMRI have shown increased perfusion in the amygdala, hippocampus, insula, but decreased perfusion in the medial prefrontal cortex (395–398).

## Discussion and Actionable Recommendations

Given the major advances in our understanding of the SPECT functional neuroimaging findings for both neurological and psychiatric conditions as iterated above, the position assumed in the wake of the TTASAAN report by both the fields of Neurology and Psychiatry do not appear tenable. As a result, the following recommendations are made for the revisions of the current policies and practices as they relate to perfusion SPECT functional neuroimaging:

1. Increase awareness of the actual current state of the art as iterated above.
2. Replace assumptions about the inferiority of SPECT neuroimaging compared to PET, fMRI, diffusion tensor imaging, and MEG neuroimaging, particularly in the areas of dementia, TBI, seizure disorders, and neuropsychiatric indications with updated comparisons as elaborated herein.
3. Foster collaboration and communication between Nuclear Medicine physicians knowledgeable about perfusion SPECT neuroimaging and neurologists, psychiatrists, and other prescribers.
4. Improve knowledge of the technical aspects of perfusion SPECT neuroimaging to improve understanding of the limitations and strengths of the procedure. This will be addressed in Part II of this two-part series (399).
5. Revise Nuclear Medicine procedure guidelines to match the current state of the art as elaborated herein. This process has already begun with the publication of the new Canadian Association of Nuclear Medicine Guidelines for Brain Perfusion Single Photon Emission Computed Tomography (SPECT) (3).
6. Revise Neurology practice guidelines to include the use of perfusion SPECT neuroimaging in the areas for which it has been shown to be most effective or on par with FDG-PET neuroimaging (e.g., seizure disorders, dementia, stroke). The case is made herein that SPECT is a potent tool in the evaluation of TBI and warrants inclusion in Neurology practice guidelines based on Level IIa evidence meeting the criteria for a Type A recommendation by the standards set forth in the TTASAAN report (1) and the moral impossibility of achieving Class I evidence.
7. Revise Neurology and Psychiatry practice guidelines to recognize the advances in the state of the art of SPECT neuroimaging in the *evaluation of* neuropsychiatric indications. As the Canadian Association of Nuclear Medicine Guidelines for Brain Perfusion Single Photon Emission Computed Tomography (SPECT) (3) describes, the diagnostic picture for all of neuroimaging is clouded due to the subjective and non-physiological bases of psychiatric diagnoses. This remains an unresolved issue.
8. Understand the basics of reading and interpreting a perfusion brain SPECT scan based on the findings summarized herein. This will be addressed in Part II of this two-part series (399).

Perfusion SPECT scans require rigorous technique and correct adjustment of the equipment. An enormous variability in SPECT images results from technical inconsistency. The number of brain SPECT procedures performed annually is relatively small, largely due to the small number of scans ordered by treating physicians. One of the reasons why brain SPECT imaging is not prescribed more often is that prior encounters with sub-optimal images resulting from poor technique and lack of experience, have produced unhelpful findings. These negative experiences deter further use of SPECT scans and leads to fewer referrals. In part because of this dynamic and the parallel academic pressure to move to PET-based procedures, the attention, experience, and interest of nuclear medicine physicians has shifted away from SPECT imaging. In addition, the wholesale bias against perfusion SPECT scans discussed heretofore has stymied the clinical growth of this inexpensive, useful, and readily available procedure. The technical aspects of correctly performing and reading these scans will be detailed in part II of this two-part series (399).

Although the focus within the nuclear medicine community has shifted toward brain PET imaging, there currently is a growing interest in brain SPECT from the field of psychiatry as it becomes more evident that these scans can be immensely helpful in developing treatment plans for certain conditions. Moreover, with the recent introduction of new camera technologies detailed in part II of this two-part series (399), it is possible to produce images that rival FDG-PET quality with lower radiation exposure and less cost.

## Conclusions

Simply put—this is not your father's Oldsmobile. The SPECT scans of the early 1990's were limited due to single-headed gamma cameras, unstable or rapidly decaying tracers, limited post-processing techniques, and absence of quantitative analysis. Twenty-five years later, SPECT scans have much greater resolution, multiple normative databases, numerous studies often with quite large sample sizes, and sophisticated post-processing software. Moreover, the introduction of a new solid-state detector system utilizing cadmium-zinc-telluride (CZT) diodes will greatly increase the resolution of SPECT and reduce the radiation dose required. These improvements will address two limitations often cited concerning SPECT. These technical aspects are detailed in Part II of this series.

The path into the future will be paved with collaborations between nuclear medicine physicians, neurologists, and psychiatrists. Hopefully, enlightened clinicians in these fields will join together to deepen their clinical experience; hence, advancing and expand our understanding of the perfusion SPECT neuroimaging correlates in neurology and neuropsychiatry yielding improved treatment outcomes as has been demonstrated in several pilot studies (109, 110, 302, 349, 400).

## Author Contributions

DP and TH conceived the manuscript. DP, TH, and SD wrote initial drafts. TH and SD prepared revisions and prepared the figures. All authors contributed to the article and approved the submitted version.

## Conflict of Interest

DP is Director of PathFinder Brain SPECT which is a clinical service corporation providing SPECT functional neuroimaging and had no research funding. He is deceased. TH is the president and principal owner of The Synaptic Space, a neuroimaging consulting firm. He is also CEO and Chairman of the Board of Neuro-Luminance Corporation, a medical service company. He is also president and principal owner of Dr. Theodore Henderson, Inc., a medical service company. He is also Vice-President of the Neuro-Laser Foundation, a non-profit organization. He is a member of and a former officer of the Brain Imaging Council Board of the Society of Nuclear Medicine and Molecular Imaging (SNMMI). Since 2017, he has served in the SNMMI Brain Imaging Outreach Working Group. Currently, he serves as president of the International Society of Applied Neuroimaging. TH has no ownership in, and receives no remuneration from, any neuroimaging company. No more than 5% of his income is derived from neuroimaging. SD is President of Good Lion Imaging LLC involved in the post-processing and display of functional brain scan data. The reviewer DA declared a past co-authorship with one of the authors TH to the handling editor.

## Publisher's Note

All claims expressed in this article are solely those of the authors and do not necessarily represent those of their affiliated organizations, or those of the publisher, the editors and the reviewers. Any product that may be evaluated in this article, or claim that may be made by its manufacturer, is not guaranteed or endorsed by the publisher.
